# Optogenetic manipulation of lysosomal physiology and autophagy-dependent clearance of amyloid beta

**DOI:** 10.1371/journal.pbio.3002591

**Published:** 2024-04-23

**Authors:** Wenping Zeng, Canjun Li, Ruikun Wu, Xingguo Yang, Qingyan Wang, Bingqian Lin, Yanan Wei, Hao Li, Ge Shan, Lili Qu, Chunlei Cang

**Affiliations:** 1 Institute on Aging and Brain Disorders, The First Affiliated Hospital of USTC, Division of Life Sciences and Medicine, University of Science and Technology of China, Hefei, Anhui, China; 2 Institute of Health and Medicine, Hefei Comprehensive National Science Center, Hefei, Anhui, China; 3 Department of Laboratory Medicine, The First Affiliated Hospital of USTC, Division of Life Science and Medicine, University of Science and Technology of China, Hefei, Anhui, China; 4 The CAS Key Laboratory of Innate Immunity and Chronic Disease, School of Basic Medical Sciences, Division of Life Sciences and Medicine, University of Science and Technology of China, Hefei, Anhui, China; 5 Neurodegenerative Disorder Research Center, University of Science and Technology of China, Hefei, Anhui, China; Rheinische Friedrich-Wilhelms-Universitat Bonn, GERMANY

## Abstract

Lysosomes are degradation centers of cells and intracellular hubs of signal transduction, nutrient sensing, and autophagy regulation. Dysfunction of lysosomes contributes to a variety of diseases, such as lysosomal storage diseases (LSDs) and neurodegeneration, but the mechanisms are not well understood. Altering lysosomal activity and examining its impact on the occurrence and development of disease is an important strategy for studying lysosome-related diseases. However, methods to dynamically regulate lysosomal function in living cells or animals are still lacking. Here, we constructed lysosome-localized optogenetic actuators, named lyso-NpHR3.0, lyso-ArchT, and lyso-ChR2, to achieve optogenetic manipulation of lysosomes. These new actuators enable light-dependent control of lysosomal membrane potential, pH, hydrolase activity, degradation, and Ca^2+^ dynamics in living cells. Notably, lyso-ChR2 activation induces autophagy through the mTOR pathway, promotes Aβ clearance in an autophagy-dependent manner in cellular models, and alleviates Aβ-induced paralysis in the *Caenorhabditis elegans* model of Alzheimer’s disease. Our lysosomal optogenetic actuators supplement the optogenetic toolbox and provide a method to dynamically regulate lysosomal physiology and function in living cells and animals.

## Abstract 2

Lysosomes are cellular degradation centers, critical for maintaining homeostasis. Selective modulation of lysosomal activity has remained a challenge to date; this study reports the development of lysosome-targeted optogenetic tools for the light-inducible manipulation of lysosomal physiology in cells and in C. elegans.

## Introduction

Lysosomes are the main degradative organelles in eukaryotic cells and are responsible for degrading and recycling various biomacromolecules delivered by endocytosis, phagocytosis, and autophagy [1]. Recent studies have found that lysosomes are also important signaling nodes and regulate various cellular processes [2,3]. Dysfunction of lysosomes has been linked to a wide variety of human disorders, including lysosomal storage diseases, cancer, and neurodegeneration, and restoration of lysosomal activity holds promise as an effective strategy for the treatment of these diseases [4,5]. Specific drugs targeting lysosomal proteins or genetic manipulation of lysosomal-related genes are common means of controlling lysosomal function. However, methods that can be used to dynamically regulate lysosomal activity are lacking, which is a disadvantage for the study of lysosome-related disease mechanisms and drug development.

Optogenetics is an emerging technique that utilizes genetically encoded light-sensitive proteins, such as rhodopsins, enzymes, phytochromes, and cryptochromes, to manipulate cellular activities with light [6–8]. In neuroscience research, optogenetics is widely used to control the activity of neurons in living cells or living animals. The distribution of ions across the neuronal cell membrane and the resulting membrane potential are critical to neuronal function, making neurons very sensitive to optogenetic modulation. Using light to control microbial opsins, such as channelrhodopsin-2 (ChR2) [9,10] or halorhodopsin (NpHR) [11], can manipulate the ion homeostasis and membrane potential of neurons and thus control the activity of these cells [12,13]. Beyond its well-known applications in neuroscience, optogenetics is extensively employed in regulating various intracellular biological processes, including but not limited to, intracellular signaling pathways [14], protein–protein interactions [15], gene expression [16], and organelle repositioning [17].

Ions across the lysosomal membrane are also unevenly distributed, which is critical for lysosomal function [18]. Most lysosomal enzymes are acid hydrolases, and their activity depends on the high concentration of H^+^ in the lysosomal lumen. Lysosomes are also important intracellular stores for metal ions such as Ca^2+^ and Fe^2+^. The storage and release of these ions are involved in the regulation of a variety of lysosomal functions [18]. The lysosomal membrane potential plays an important role in maintaining lysosomal ion homeostasis. In addition, lysosomes also have specific forms of electrical excitability [19], although the functional significance of this excitability is unclear. Disruption of ion homeostasis is an important cause of lysosome-related disease. A variety of ion channels are expressed on the lysosomal membrane. Previous studies have found that the activity of these channels strongly regulates lysosomal function [20]. Most lysosomal ion channels lack specific drugs, so interfering with the expression of these ion channels or making mutations through genetic engineering techniques are common methods to control their activity.

Given the importance of lysosomal ion homeostasis and membrane potential to lysosomal function, we hypothesized that optogenetics could be used to manipulate the physiological states and activity of lysosomes. By inserting lysosomal signal peptides, we successfully targeted 3 commonly used optogenetic actuators, NpHR3.0, ArchT, and ChR2, to the lysosomal membrane. By photoactivating these actuators, we achieved optogenetic manipulation of lysosomal physiology and activity in living cells and *Caenorhabditis elegans* (*C*. *elegans*) and examined the effect of lysosomal photoactivation on amyloid-beta (Aβ) clearance in Alzheimer’s disease (AD) models.

## Results

### Targeting optogenetic actuators to lysosomes

**Fig 1:**
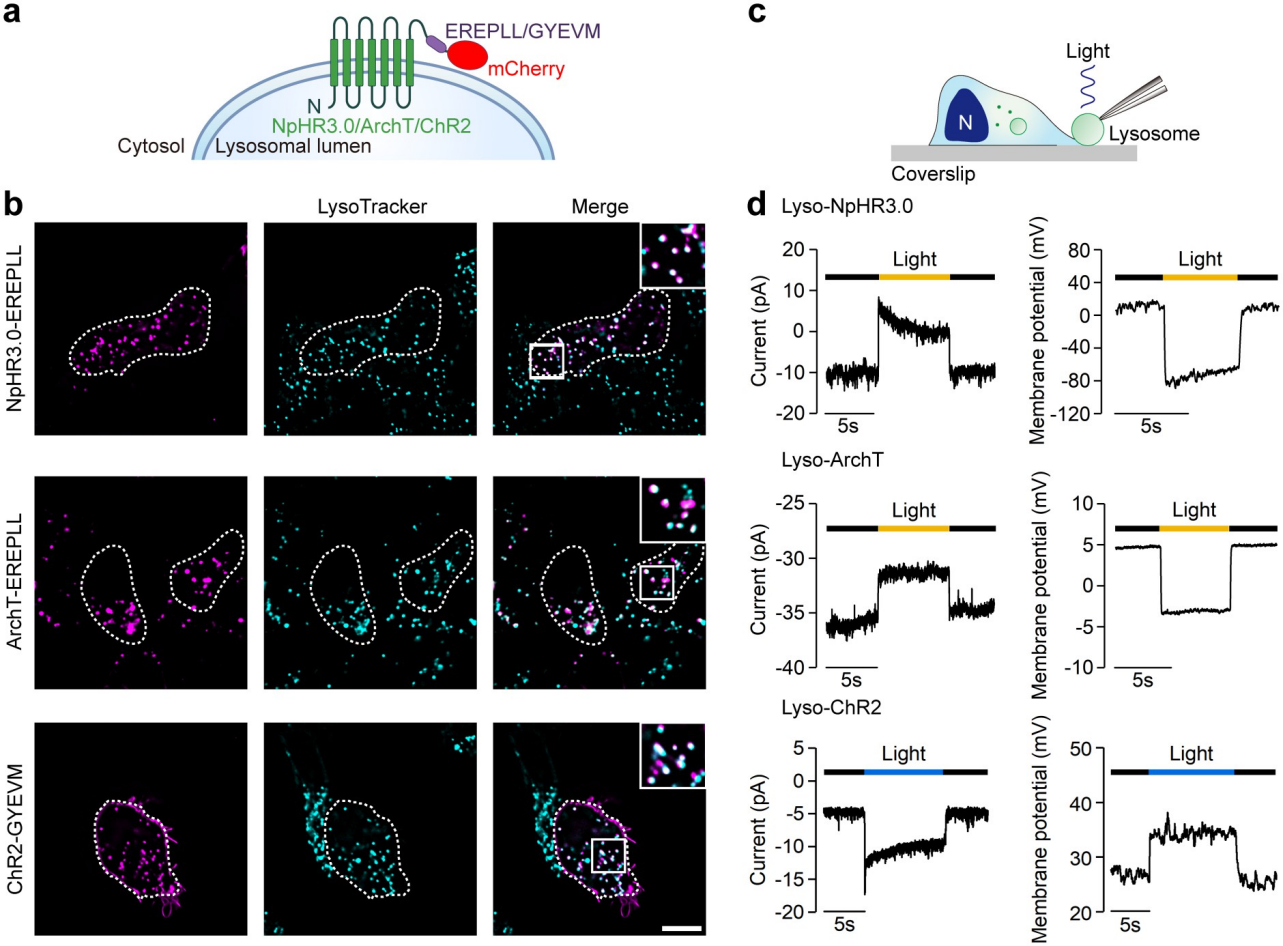
Localizations of the lysosome-targeting optogenetic actuators. (a) Design and membrane topology of the lyso-NpHR3.0/ArchT/ChR2. (b) Colocalization of C-terminally mCherry-tagged lyso-NpHR3.0 (upper), lyso-ArchT (middle), and lyso-ChR2 (lower) with LysoTracker Green in HEK293T cells. A magnified image of the white boxed area is shown in the upper right corner of the merged image. Scale bar, 10 μm. (c) Schematic diagram of the section view of the whole-lysosome patch clamp recording. An enlarged lysosome is extruded through the cellular incision and recorded with glass pipette under light stimulation. The dark blue represents nucleus. (d) Lysosomal membrane currents (left column) and membrane potentials (right column) evoked by brief corresponding light applications in lyso-NpHR3.0- (upper), lyso-ArchT- (middle), or lyso-ChR2-expressing (lower) HEK293T cells. The data underlying this figure can be found in S1 Data.

Lysosomal membrane proteins often utilize 2 types of sorting signals to direct their lysosomal localization, namely, dileucine-based motifs (DXXLL or [DE]XXXL[LI]) and tyrosine-based motifs (YXXØ) [21]. To introduce light sensitivity to lysosomes, we first added the dileucine-based sorting motif EREPLL of the lysosomal transporter protein PQLC2 [22] to the C-terminus of 3 commonly used light-sensitive proteins, NpHR3.0, ArchT, and ChR2. In addition, the fluorescent protein mCherry was fused to the C-terminus of the motif to indicate protein expression and localization (Fig 1a). Both modified NpHR3.0 and ArchT were distributed in puncta within the cells and colocalized well with the lysosome marker LysoTracker Green in HEK293T cells, indicating successful lysosomal localization (Fig 1b). Enlarging lysosomes by treating cells with vacuolin-1 allowed us to clearly see that the engineered NpHR3.0 and ArchT were expressed on the lysosomal membrane (S1 Fig). However, EREPLL-modified ChR2 showed little colocalization with LysoTracker (S2a Fig). Attempts to enhance lysosomal targeting with doubled signals or the PQLC2 C-terminus (containing the targeting signal), creating ChR2-2×EREPLL and ChR2-PQLC2-C, were unsuccessful (S2b and S2c Fig). We then tried another type of signal, YXXØ, by inserting CD63’s GYEVM [21,23] or LAMP-2a’s GYEQF [21] into ChR2’s C-terminus (Fig 1a), yielding ChR2-GYEVM and ChR2-GYEQF. Both constructs displayed intracellular puncta which strong colocalized with lysosomes, despite some presence on the plasma membrane (Fig 1b and S2d Fig). The ChR2-GYEVM was used for subsequent experiments. We named EREPLL-modified NpHR3.0 and ArchT and GYEVM-modified ChR2 lyso-NpHR3.0, lyso-ArchT, and lyso-ChR2, respectively.

To determine whether the lysosome-targeting optogenetic actuators could function normally, we performed whole-lysosome patch clamp recording in HEK293T cells (Fig 1c). Illumination with yellow light elicited lysosomal membrane hyperpolarization and outward currents in lysosomes expressing lyso-NpHR3.0 or lyso-ArchT (Fig 1d). Here, the lysosomal membrane potential (Ψ) is defined as V_cytosol_—V_lumen_, and the outward current denotes the movement of cations out of the cytosol into the lumen or the movement of anions out of the lumen. Lysosomal membrane depolarization and inward currents were observed in lysosomes expressing lyso-ChR2 upon blue light illumination (Fig 1d). These data demonstrated that EREPLL- or GYEVM-modified optogenetic actuators could successfully target lysosomes and evoke lysosomal membrane potential and current changes in response to light.

### Light-dependent control of lysosomal pH

**Fig 2:**
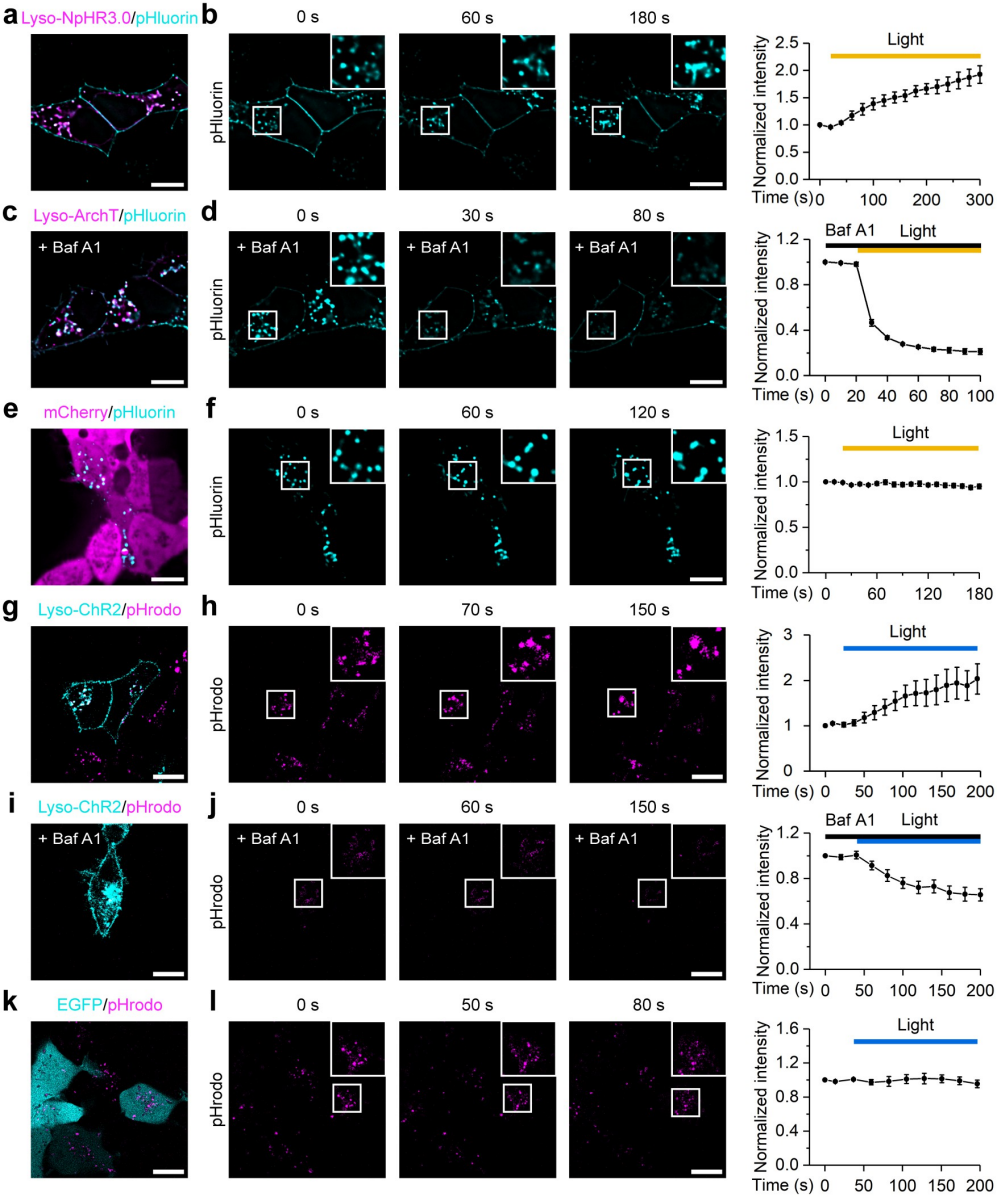
Lysosome-targeted optogenetic actuators regulate lysosomal pH. (a) Colocalization of mCherry-tagged lyso-NpHR3.0 (magenta) with lyso-pHluorin (cyan) in HEK293T cells. (b) pHluorin signals acquired before (time point 0 s) and during (60 and 180 s) lyso-NpHR3.0 activation (left), and the time plot of normalized (to 0 s) pHluorin signals (right, *n* = 5). (c) Colocalization of mCherry-tagged lyso-ArchT (magenta) with lyso-pHluorin (cyan) in Baf A1-pretreated HEK293T cells. (d) pHluorin signals of Baf A1-pretreated HEK293T cells acquired before (time point 0 s) and during (30 and 80 s) lyso-ArchT activation (left), and the time plot of normalized (to 0 s) pHluorin signals (right, *n* = 3). (e) Confocal images of HEK293T cells cotransfected with mCherry (magenta) and lyso-pHluorin (cyan). (f) pHluorin signals acquired before (time point 0 s) and during (60 and 120 s) 594 nm light stimulation (left), and the time plot of normalized (to 0 s) pHluorin signals (right, *n* = 5). (g) Colocalization of EGFP-tagged lyso-ChR2 (cyan) with pHrodo Red Dextran (magenta) in HEK293T cells. (h) pHrodo signals acquired before (time point 0 s) and during (70 s and 150 s) lyso-ChR2 activation (left), and the time plot of normalized (to 0 s) pHrodo signals (right, *n* = 4). (i) Colocalization of EGFP-tagged lyso-ChR2 (cyan) with pHrodo Red Dextran (magenta) in Baf A1-pretreated HEK293T cells. (j) pHrodo signals of Baf A1-pretreated HEK293T cells acquired before (time point 0 s) and during (60 s and 150 s) lyso-ChR2 activation (left), and the time plot of normalized (to 0 s) pHrodo signals (right, *n* = 5). (k) Confocal images of HEK293T cells transfected with EGFP (cyan) and incubated with pHrodo Red Dextran (magenta). (l) pHrodo signals acquired before (time point 0 s) and during (50 s and 80 s) 488 nm light stimulation (left), and the time plot of normalized (to 0 s) pHrodo signals (right, *n* = 4). Scale bars, 10 μm. Data are shown as mean ± SEM. The data underlying this figure can be found in S1 Data.

The acidic pH in the lysosomal lumen is crucial for hydrolase activity and lysosomal degradation. Lysosomal pH is mainly established by V-ATPase and regulated by various lysosomal ion channels [19,24,25], so activation of lysosome-targeted optogenetic actuators is likely to affect lysosomal pH. To test this hypothesis, we utilized lyso-pHluorin, a pH-sensitive fluorescent protein targeting lysosomes, to monitor lysosomal pH in real time. Both lyso-NpHR3.0 and lyso-ArchT showed strong colocalization with lyso-pHluorin in HEK293T cells (Fig 2a and 2c). Activation of lyso-NpHR3.0 with 594 nm light caused a time-dependent increase in pHluorin signals, indicating an increase in lysosomal pH (Fig 2b). Since ArchT is an outward proton pump when expressed on the plasma membrane [26], we speculated that lyso-ArchT would pump protons into the lysosomal lumen and thus decrease lysosomal pH. Since the pH of lysosomes is already low under normal conditions, which may make the effect of lyso-ArchT difficult to detect, we first used the V-ATPase inhibitor bafilomycin A1 (Baf A1) to increase the lysosomal pH. As expected, preincubation with 1 μM Baf A1 for 2 h significantly enhanced the fluorescence of pHluorin, and subsequent activation of lyso-ArchT by 594 nm light resulted in a rapid decrease in the pHluorin signal (Fig 2d), reflecting fast lysosomal acidification. This is consistent with the results obtained from lysosome-targeted Arch3 in a previous study [27]. The decrease in pHluorin fluorescence was not due to 594 nm photostimulation or photobleaching during prolonged imaging, as the pHluorin signals did not change significantly in HEK293T cells expressing mCherry and lyso-pHluorin during the same imaging procedure (Fig 2e and 2f).

Since the excitation spectra of pHluorin and ChR2 overlap, we monitored the effect of lyso-ChR2 activation on lysosomal pH using pHrodo Red Dextran, a pH-sensitive red fluorescent dye conjugated to dextran that reaches lysosomes via endocytosis. Since ChR2 is highly permeable to protons [10], we speculated that activation of lyso-ChR2 might cause leakage of H^+^ from the lysosomal lumen to raise lysosomal pH. Surprisingly, we found that the pHrodo signal increased upon activation of lyso-ChR2 with 488 nm light, indicating a decrease in lysosomal pH (Fig 2g and 2h). After inhibiting V-ATPase with Baf A1, the pHrodo signals decreased upon exposure to 488 nm light (Fig 2i and 2j), suggesting that the decrease in the pH of lysosomes caused by lysosomal-ChR2 activation may be achieved through indirect activation of V-ATPase. Likewise, the decrease in the pHrodo signal in Fig 2j was not caused by light stimulation or signal acquisition (Fig 2k and 2l). Taken together, our lysosome-targeted optogenetic actuators enabled regulation of lysosomal pH with light.

### Light-dependent control of lysosomal hydrolase activity and degradation

Most lysosomal hydrolases are pH-sensitive, so changes in lysosomal pH can affect the activity of these hydrolases and the degradative capacity of lysosomes. We next determined the effect of photoactivation of the 3 lysosome-targeted optogenetic actuators on lysosomal hydrolase activity and degradation. To avoid spectral overlap between the fluorescent dyes used for detection and fluorescent proteins that label optogenetic actuators, we constructed stable HEK293T cell lines expressing lysosome-targeted optogenetic actuators without fluorescent labeling. Patch clamp recordings showed normal activities of lyso-NpHR3.0, lyso-ArchT, and lyso-ChR2 without fluorescent labeling (S3 Fig).

**Fig 3:**
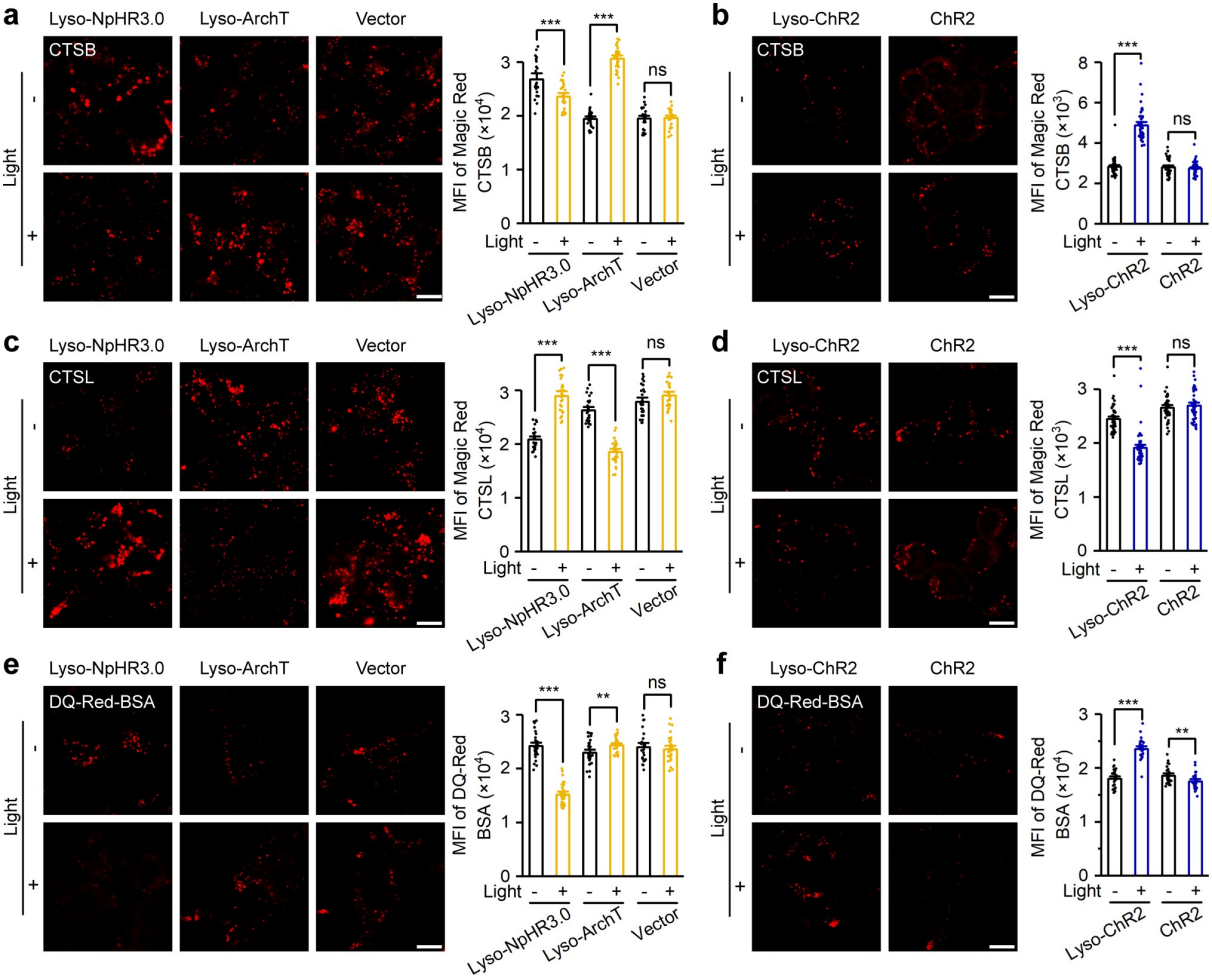
Lysosome-targeted optogenetic actuators regulate lysosomal enzyme activity and degradation. (a) Magic Red CTSB staining of HEK293T cells stably expressing lyso-NpHR3.0, lyso-ArchT, or vector, with or without yellow light (left), and the MFI of Magic Red CTSB (right, *n* = 22, 28, 28, 28, 28, and 29 for bars from left to right). (b) Magic Red CTSB staining of HEK293T cells stably expressing lyso-ChR2 or ChR2, with or without blue light (left), and the MFI of Magic Red CTSB (right, *n* = 50, 50, 44, and 50 for bars from left to right). (c) Magic Red CTSL staining of HEK293T cells stably expressing lyso-NpHR3.0, lyso-ArchT, or vector, with or without yellow light (left), and the MFI of Magic Red CTSL (right, *n* = 28, 28, 28, 31, 28, and 28 for bars from left to right). (d) Magic Red CTSL staining of HEK293T cells stably expressing lyso-ChR2 or ChR2, with or without blue light (left), and the MFI of Magic Red CTSL (right, *n* = 58, 63, 50, and 49 for bars from left to right). (e) Representative images of HEK293T cells stably expressing lyso-NpHR3.0, lyso-ArchT, or vector, incubated with DQ-Red-BSA, with or without yellow light (left), and the MFI of DQ-Red-BSA (right, *n* = 30, 31, 31, 28, 27, and 29 for bars from left to right). (f) Representative images of HEK293T cells stably expressing lyso-ChR2 or ChR2, incubated with DQ-Red-BSA, with or without blue light (left), and the MFI of DQ-Red-BSA (right, *n* = 29, 29, 29, and 28 for bars from left to right). Scale bars, 10 μm. Data are shown as mean ± SEM. ** *P* < 0.01, *** *P* < 0.001; ns, not significant. The data underlying this figure can be found in S1 Data. MFI, mean fluorescence intensity.

Using cathepsin B (CTSB) and cathepsin L (CTSL) as representatives, we investigated the effect of optogenetic manipulation on lysosomal hydrolase activity. HEK293T cells expressing lysosomal actuators were stained with Magic Red CTSB/CTSL and illuminated for 6 h with yellow light (for lyso-NpHR3.0 and lyso-ArchT; 2.15 mW/cm^2^, 5 Hz with 80 ms light pulse) or blue light (for lyso-ChR2; 5.6 mW/cm^2^, 10 Hz with 50 ms light pulse). Such light stimulations did not alter cell viability (S4 Fig). Photoactivation of lyso-NpHR3.0 decreased the fluorescence signals of Magic Red CTSB and increased the Magic Red CTSL signals (Fig 3a and 3c), indicating a decrease in CTSB activity and an increase in CTSL activity. Photoactivation of lyso-ArchT by the same light stimulation gave the opposite results, namely, increased CTSB activity and decreased CTSL activity (Fig 3a and 3c), which may be attributed to the different regulation of lysosomal pH by the activation of the 2 optogenetic actuators (Fig 2a–2d). Light stimulation of HEK293T cells expressing the empty vector, as controls for lyso-NpHR3.0 and lyso-ArchT, did not cause changes in CTSB or CTSL activity (Fig 3a and 3c). Similar to lyso-ArchT, illumination of HEK293T cells stably expressing lyso-ChR2 by blue light for 6 h (5.6 mW/cm^2^, 10 Hz with 50 ms light pulse) resulted in an increase in CTSB activity and a decrease in CTSL activity (Fig 3b and 3d). Meanwhile, light-controlled changes in enzymatic activity were not observed in cells expressing PM-targeted ChR2 (Fig 3b and 3d).

Using DQ-Red BSA, a probe that yields red fluorescent products after being hydrolyzed in lysosomes, we evaluated the effect of optogenetic manipulation on lysosomal degradation capacity. Activation of lyso-NpHR3.0 by light decreased BSA degradation, while activation of lyso-ArchT or lyso-ChR2 increased BSA degradation (Fig 3e and 3f). Thus, our lysosomal optogenetic tools confer photosensitivity to lysosomal enzymatic activity and lysosomal degradation.

### Light-dependent control of lysosomal Ca^2+^ dynamics

Lysosomes are important intracellular Ca^2+^ stores, and the storage and release of lysosomal Ca^2+^ are of great significance to lysosomal physiology and signal transduction [28]. We next asked whether our lysosome-targeted optogenetic tools could control lysosomal Ca^2+^ dynamics.

**Fig 4:**
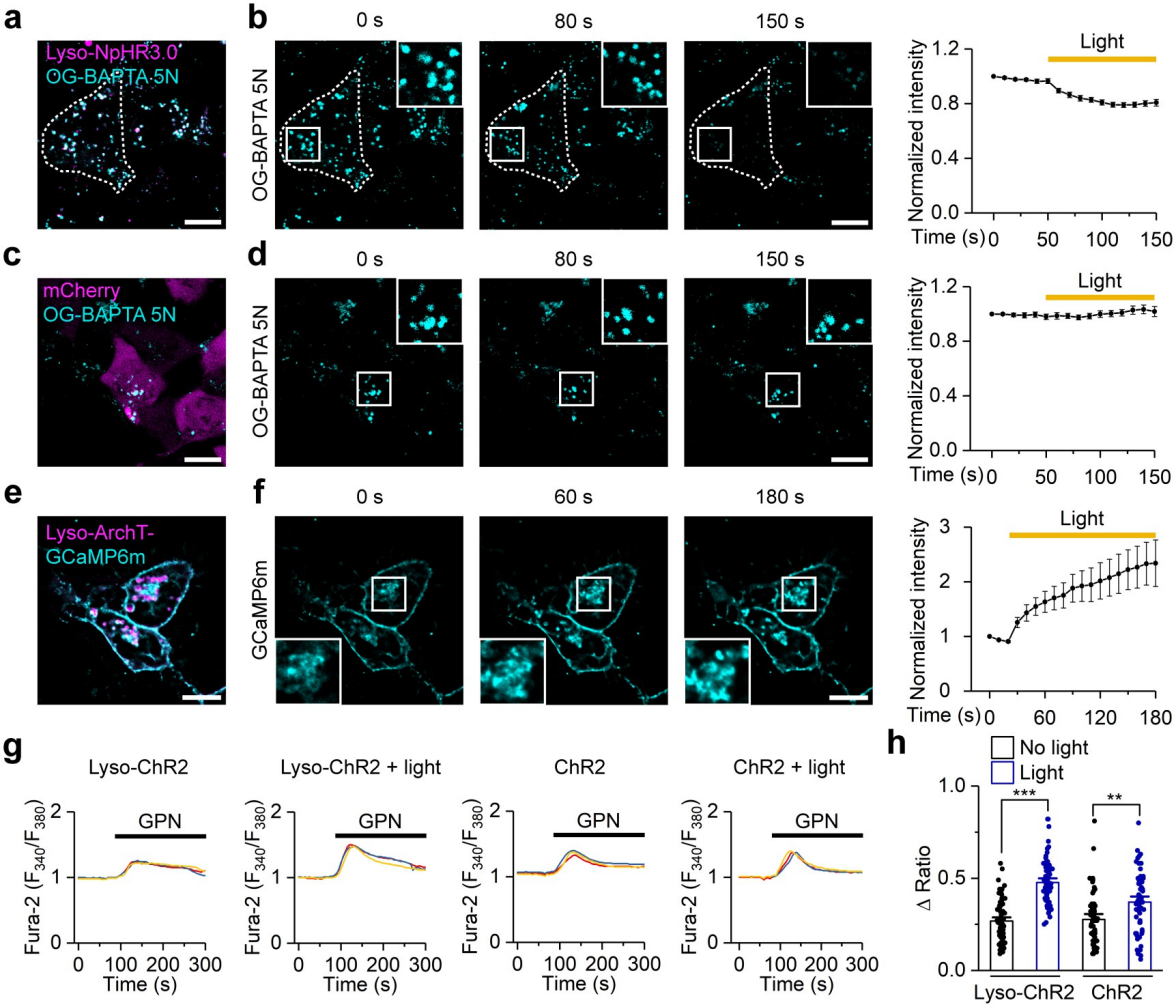
Lysosome-targeted optogenetic actuators regulate lysosomal Ca^2+^ dynamics. (a) Colocalization of mCherry-tagged lyso-NpHR3.0 (magenta) with OG-BAPTA 5N (cyan) in HEK293T cells. (b) OG-BAPTA 5N signals acquired before (time point 0 s) and during (80 and 150 s) lyso-NpHR3.0 activation (left), and the time plot of normalized (to 0 s) OG-BAPTA 5N signals (right, *n* = 14). (c) Fluorescence images of HEK293T cells transfected with mCherry (magenta) and incubated with OG-BAPTA 5N (cyan). (d) OG-BAPTA 5N signals acquired before (time point 0 s) and during (80 and 150 s) 594 nm light stimulation (left), and the time plot of normalized (to 0 s) OG-BAPTA 5N signals (right, *n* = 11). (e) Representative images of HEK293T cells transfected with lyso-ArchT-GCaMP6m-mCherry. (f) GCaMP6m signals acquired before (time point 0 s) and during (60 and 180 s) 594 nm light stimulation (left), and the time plot of normalized (to 0 s) GCaMP6m signals on lysosomes (right, *n* = 3). (g) Representative pictures of [Ca^2+^]_i_ intensity before and after GPN-induced lysosomal store depletion measured with Fura-2 (F_340_/F_380_) in HEK293T cells stably expressing lyso-ChR2 or ChR2, with or without blue light. (h) GPN-induced changes in Fura-2 fluorescence ratio F_340_/F_380_ (Δratio) in HEK293T cells with different treatments as indicated. *n* = 73, 69, 56, and 65 for bars from left to right. Scale bars, 10 μm. Data are presented as mean ± SEM. ** *P* < 0.01, *** *P* < 0.001. The data underlying this figure can be found in S1 Data.

To examine the effect of lyso-NpHR3.0 activation on lysosomal Ca^2+^ contents, we used OG-BAPTA-5N as an indicator, which could enter the lysosome by endocytosis. The fluorescence signals of OG-BAPTA-5N gradually decreased under continuous 594 nm light stimulation (Fig 4a and 4b), while no significant changes were detected in the control cells expressing mCherry (Fig 4c and 4d).

We employed GCaMP6m, a Ca^2+^-sensitive fluorescent protein, to detect lysosomal Ca^2+^ release elicited by lyso-ArchT activation. The sequence of GCaMP6m was inserted into lyso-ArchT at a position between the lysosome-targeting signal EREPLL and the red fluorescent protein mCherry, and the resulting fusion protein lyso-ArchT-GCaMP6m was transfected into HEK293T cells and stimulated by 594 nm light (Fig 4e). As shown in Fig 4f, the fluorescence intensity of GCaMP6m gradually increased with the photoactivation of lyso-ArchT, indicating an increase in lysosomal Ca^2+^ release.

Due to overlapping excitation spectra, the above 2 calcium probes could not be used to test the effect of lyso-ChR2 on lysosomal calcium. Therefore, we used glycyl-L-phenylalanine 2-naphthylamide (GPN) to release lysosomal Ca^2+^ and used Fura-2 to detect changes in cytosolic Ca^2+^ concentration to indirectly estimate lysosomal calcium content. After 6 h of blue light stimulation, GPN-induced Fura-2 signaling was significantly increased in lyso-ChR2-expressing HEK293T cells, indicating an increase in lysosomal calcium content (Fig 4g and 4h). In summary, the above results showed that the 3 lysosome-targeted optogenetic tools we constructed could control lysosomal Ca^2+^ dynamics.

### Lyso-ChR2 activation induces autophagy through the mTOR pathway

Autophagy is a lysosomal degradation pathway that clears unnecessary or dysfunctional components to maintain cellular homeostasis. In addition to being the endpoint of autophagic flux, lysosomes also regulate upstream processes of autophagy. Therefore, we next determined the potential of lysosomal optogenetics in regulating autophagy.

**Fig 5:**
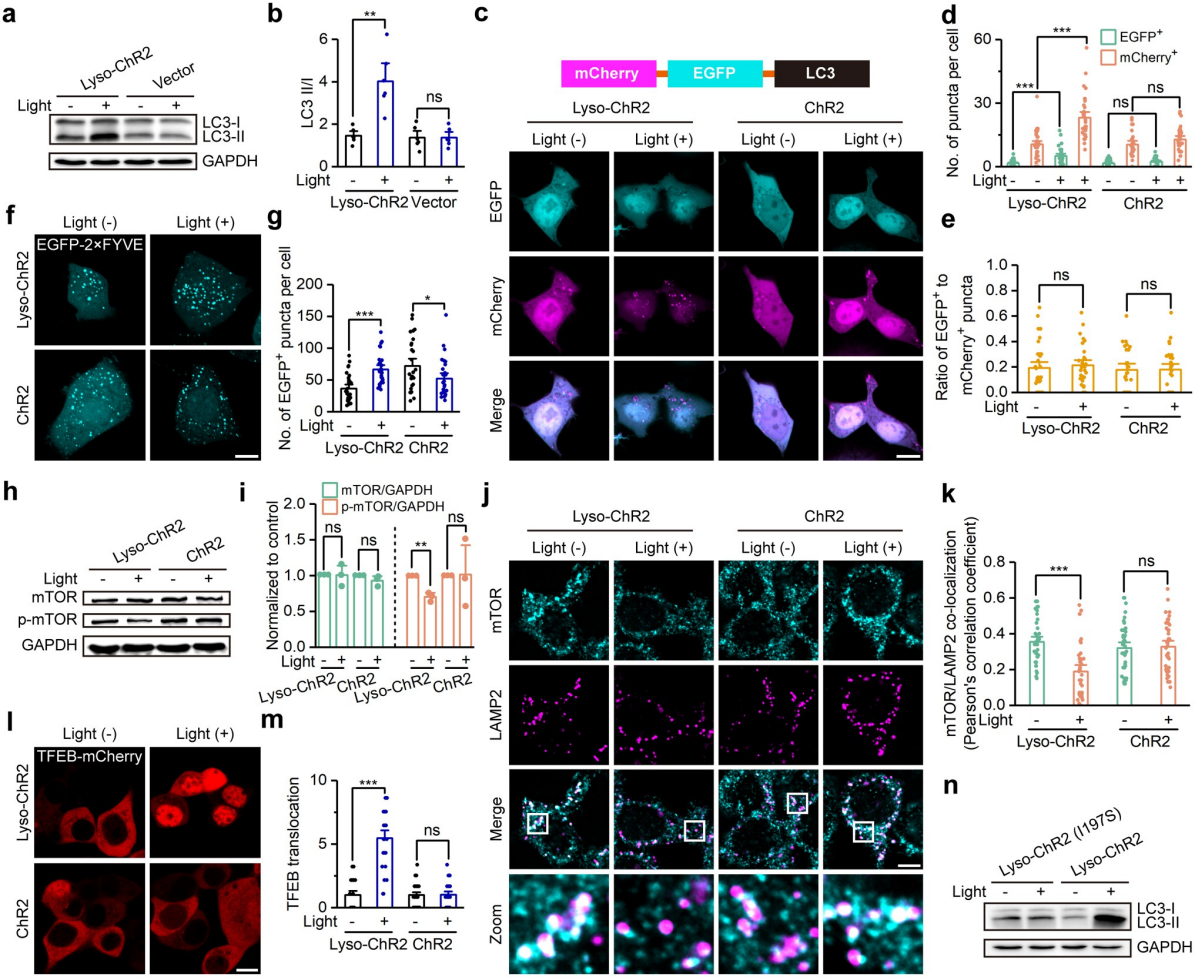
Lyso-ChR2 activation induces autophagy through the mTOR pathway. (a) Immunoblotting analysis of the protein levels of 2 forms of LC3 (LC3-I and LC3-II) in HEK293T cells stably expressing lyso-ChR2 or empty vector, with or without 6 h blue light stimulation. (b) The quantified ratio of LC3-II to LC3-I in a. *n* = 6. (c) Autophagosome-lysosome fusion as assayed with mCherry- and GFP-tagged LC3 (tandem tag, illustrated) transfected into HEK293T cells stably expressing lyso-ChR2 or ChR2, with or without blue light stimulation. Cyan puncta (top panels) represent autophagosomes before fused with lysosomes, due to low EGFP activity in autolysosomes, and magenta puncta (middle panels) represent total autophagosomes before and after phagosome-lysosome fusion. Scale bar, 10 μm. (d) Averaged numbers of EGFP and mCherry puncta upon treatments as indicated. *n* = 36, 36, 33, 33, 30, 30, 30, and 30 for bars from left to right. (e) Numbers of EGFP-positive puncta normalized to those of mCherry-positive puncta. *n* = 36, 33, 30, and 30 for bars from left to right. (f) Fluorescence images of lyso-ChR2- or ChR2-expressing HeLa cells transiently transfected with EGFP-2×FYVE, with or without 6 h blue light stimulation. Scale bar, 10 μm. (g) Statistics of the number of EGFP-2×FYVE puncta per cell in f. *n* = 29, 29, 28, and 29 for bars from left to right. (h) Immunoblotting analysis of mTOR and p-mTOR protein levels in HEK293T cells stably expressing lyso-ChR2 or ChR2, with or without 6 h blue light stimulation. (i) Quantification of mTOR/GAPDH and p-mTOR/GAPDH in h normalized to no light control. *n* = 3. (j) Representative immunofluorescence images showing colocalization of mTOR and LAMP2 in HEK293T cells stably expressing either lyso-ChR2 or PM-targeted ChR2, with or without 6 h light stimulation. Scale bar, 10 μm. (k) The quantification of colocalization is presented as Pearson correlation coefficient. (l) Fluorescence images of lyso-ChR2- or ChR2-expressing HEK293T cells transiently transfected with TFEB-mCherry, with or without 6 h blue light stimulation. Scale bar, 10 μm. (m) Statistics of TFEB nuclear translocation in j. *n* = 28, 28, 37, and 31 for bars from left to right. (n) Immunoblotting analysis of the protein levels of LC3B in HEK293T cells stably expressing either lyso-ChR2 (I197S) or lyso-ChR2, with or without 6 h blue light stimulation. Data are shown as mean ± SEM. * *P* < 0.05, ** *P* < 0.01, *** *P* < 0.001; ns, not significant. The data underlying this figure can be found in S1 Data.

After 6 h of blue light stimulation, we found that the LC3-II/LC3-I ratio, an important indicator of autophagy activity, was significantly increased in HEK293T cells expressing lyso-ChR2 but not in control cells transfected with empty vector (Fig 5a and 5b). Similar results were observed in HeLa and SH-SY5Y cells (S5a and S5b Fig). These results suggest that activation of lyso-ChR2 regulates autophagy. However, no changes in the LC3-II/LC3-I ratio were observed in HEK293T cells expressing lyso-NpHR3.0 or lyso-ArchT after 6 h of yellow light stimulation (S5c and S5d Fig). The LC3-II/LC3-I ratio elicited by lyso-ChR2 gradually increased with increasing time, intensity, or pulse width of the light stimulation (S5e–S5g Fig), suggesting that we could precisely control the autophagic activity of cells by adjusting the illumination parameters.

The increase in the LC3-II/LC3-I ratio induced by lyso-ChR2 activation could be due to increased autophagic stimulation or decreased autophagosome-lysosome fusion. To better study the effect of lyso-ChR2 activation on autophagy, we monitored autophagic flux using tandem fluorescent-tagged LC3 (mCherry-EGFP-LC3, Fig 5c), which can distinguish autophagosomes from acidified autolysosomes based on the different pH stabilities of mCherry and EGFP [29,30]. To this end, we transiently transfected mCherry-EGFP-LC3 into HEK293T cell lines stably expressing lyso-ChR2 or ChR2 and quantified the number of green and red puncta (shown in the figures as cyan and magenta, respectively, to ensure accessibility for readers with color blindness). As shown in Fig 5c–5e, photoactivation of lyso-ChR2 significantly increased the number of both EGFP^+^ and mCherry^+^ puncta, while the ratio of the number of EGFP^+^ to mCherry^+^ puncta remained unchanged, indicating that lyso-ChR2 activation promoted autophagic flux rather than inhibited autophagosome-lysosome fusion. As a control, the number of EGFP^+^ and mCherry^+^ puncta in cells expressing PM-targeted ChR2 did not change after photostimulation (Fig 5c–5e). A key event in the initiation of autophagy is the synthesis of phosphatidylinositol 3-phosphate (PI3P) [31]. Therefore, we used EGFP-2×FYVE, a fluorescent probe that binds PI3P [32,33], to monitor the initiation of autophagy. The number of EGFP-2×FYVE puncta was increased after blue light illumination in lyso-ChR2-expressing HeLa cells but not in ChR2-expressing cells (Fig 5f and 5g), indicating that photoactivation of lyso-ChR2 promotes autophagy initiation.

Next, we investigated the mechanism by which lyso-ChR2 activation induces autophagy. mTOR, a key signaling molecule linking lysosomes and autophagy, was first examined. Activation of lyso-ChR2 by blue light did not affect mTOR expression but reduced mTOR phosphorylation (Fig 5h and 5i), indicating a decrease in mTOR activity. Meanwhile, dissociation of mTOR from lysosomes was observed following light stimulation of lyso-ChR2 (Fig 5j and 5k). Moreover, light illumination of lyso-ChR2 promoted nuclear translocation of transcription factor EB (TFEB) (Fig 5l and 5m), a master transcriptional regulator of lysosome biogenesis and autophagy [34]. Phosphorylation by mTOR complex 1 (mTORC1) inactivates TFEB and confines it to the cytoplasm. Activation of lyso-ChR2 inhibited the activity of mTOR, thereby releasing the inhibition of TFEB, which in turn promoted autophagy and lysosomal biogenesis. In fact, we observed an increase in the number of lysosomes after lyso-ChR2 activation (S6 Fig). Furthermore, a lysosome-targeted ChR2 mutant, lyso-ChR2 (I197S) (S7 Fig), which has minimal ion conductance [35], failed to induce autophagy under identical light stimulation (Fig 5n). This suggests that the observed inhibition of mTOR and activation of autophagy are reliant on alterations in lysosomal ion homeostasis caused by light activation of lyso-ChR2. Altogether, our results suggest that activation of lyso-ChR2 with light induces autophagy through the mTOR signaling pathway.

### Lyso-ChR2 activation promotes β-amyloid clearance and alleviates the phenotype of Alzheimer’s disease in *C*. *elegans*

**Fig 6:**
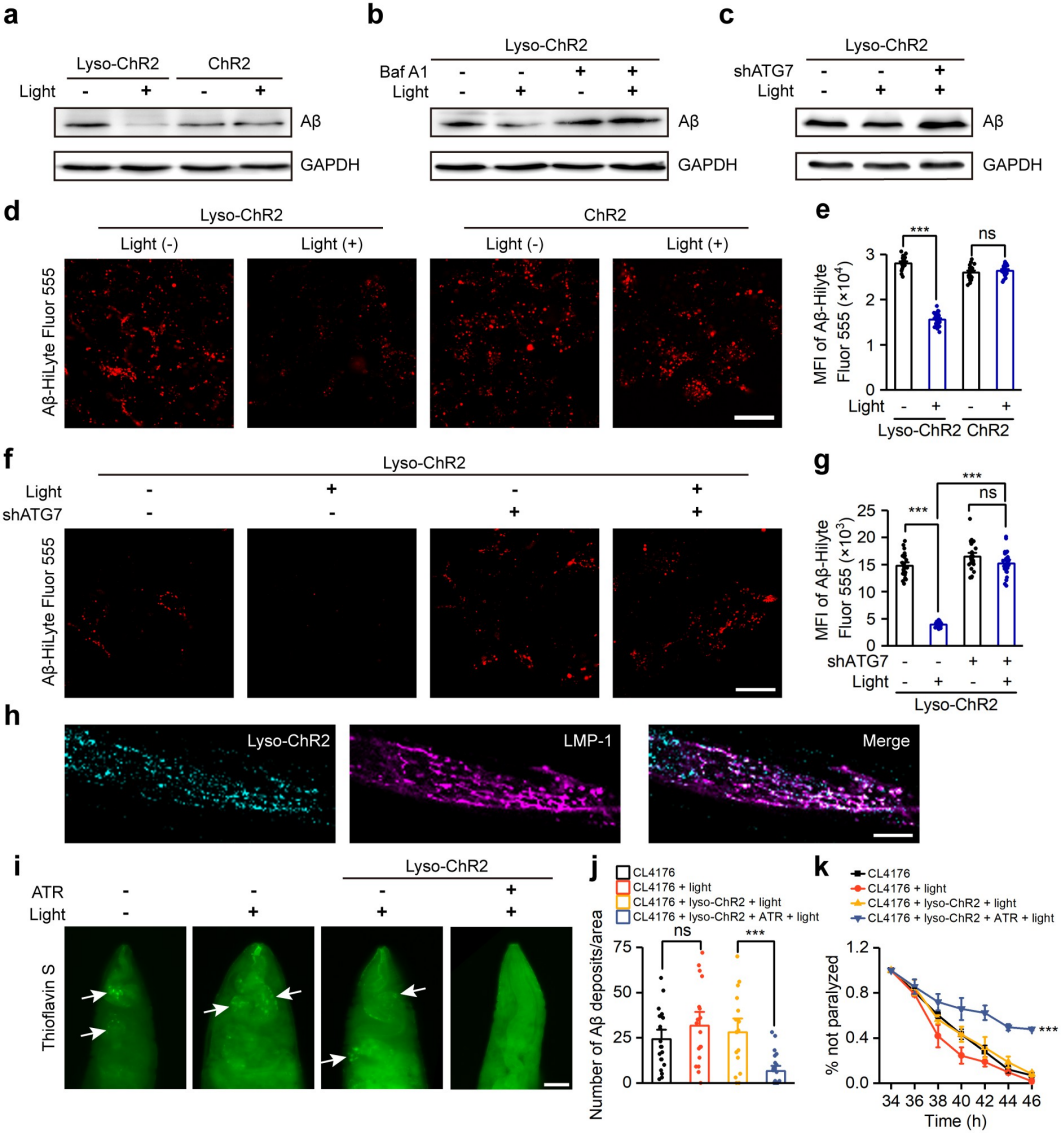
Lyso-ChR2 activation promotes Aβ clearance and alleviates the paralytic phenotype of AD in *C*. *elegans*. (a) Aβ protein levels after transient transfection of APPswe in HEK293T cells stably expressing lyso-ChR2 or ChR2, with or without blue light. (b and c) Aβ protein levels in HEK293T cell model of AD with stable expression of lyso-ChR2 in response to Baf A1 treatment (b) or *Atg7* knockdown (c), with or without blue light. (d) Representative images of Aβ-HiLyte Fluor 555 loaded into lyso-ChR2- or ChR2-expressing HEK293T cells, with or without blue light. Scale bar, 20 μm. (e) MFI of Aβ-HiLyte Fluor 555 in cells shown in d. *n* = 23, 34, 22, and 23 for bars from left to right. (f) Fluorescence images of lyso-ChR2-expressing HEK293T cells loaded with Aβ-HiLyte Fluor 555 in response to *Atg7* knockdown, with or without blue light. Scale bar, 20 μm. (g) MFI of Aβ-HiLyte Fluor 555 in cells shown in f. *n* = 25, 25, 29, and 27 for bars from left to right. (h) Colocalization of GFP-tagged lyso-ChR2 (cyan) with RFP-tagged LMP-1 (magenta) both driven by the *myo-3* promoter, in *C*. *elegans* muscle cells. Scale bar, 10 μm. (i) Representative images of *C*. *elegans* with thioflavin S staining in CL4176 strains with or without light, and CL4176 strains expressing lyso-ChR2 and fedding with or without ATR under light. Arrows indicate Aβ deposits. Scale bar, 10 μm. (j) The number of Aβ deposits in the head region/anterior area of the worms in i. *n* = 21, 18, 17, and 22 worms for bars from left to right. (k) After 34 h of Aβ induction, the paralysis rates of nematode CL4176 were assessed with the indicated treatments. *n* = 3. Data are shown as mean ± SEM. * *P* < 0.05, *** *P* < 0.001; ns, not significant. The data underlying this figure can be found in S1 Data. AD, Alzheimer’s disease; MFI, mean fluorescence intensity.

Impaired autophagy is present in many neurodegenerative diseases, such as AD. Studies have shown that induction of autophagy can clear Aβ, thereby alleviating the phenotype of AD [36–38]. Therefore, we asked whether photoactivation of lyso-ChR2 could promote Aβ clearance and alleviate the AD phenotype by inducing autophagy. To this end, we constructed a cellular AD model by overexpressing a human 695 amino acid isoform of amyloid precursor protein carrying the Swedish mutation (APPswe) [39] in HEK293T cells stably expressing lyso-ChR2 or ChR2. Stimulation with blue light for 12 h resulted in a significant reduction in Aβ levels in HEK293T cells expressing lyso-ChR2 but not in control cells expressing ChR2 (Fig 6a). Inhibition of autophagy with Baf A1 (Fig 6b) or by knocking down the essential autophagy gene *Atg7* (Fig 6c, S8a and S8b Fig) abrogated the Aβ reduction caused by lyso-ChR2 activation. To further validate the effect of lyso-ChR2 on intracellular Aβ clearance, a HiLyte Fluor 555-labeled Aβ peptide (Aβ-HiLyte Flour 555) that enters cells by endocytosis was used. HEK293T cells stably expressing lyso-ChR2 or ChR2 were preloaded with Aβ-HiLyte Flour 555 for 12 h and then illuminated with blue light for 12 h. The fluorescent signal of Aβ-HiLyte Flour 555 was considerably reduced in lyso-ChR2-expressing cells compared with ChR2-expressing cells (Fig 6d and 6e) but not in *Atg7* knockdown cells (Fig 6f and 6g). Furthermore, ELISA analysis of Aβ in the supernatant showed that lyso-ChR2 activation reduced the secretion of Aβ, which was restored by knockdown of *Atg7* (S8c Fig). These results suggest that activation of lyso-ChR2 promotes intracellular Aβ clearance and reduces extracellular Aβ release in an autophagy-dependent manner.

Next, we applied lysosomal optogenetics to living animals. A transgenic *C*. *elegans* strain, CL4176, was used to examine the effect of lyso-ChR2 photoactivation on Aβ clearance and pathology induced by Aβ accumulation. CL4176 worms carry a temperature-inducible Aβ transgene in muscle cells, resulting in a paralytic phenotype upon temperature elevation experiments [40]. We introduced lyso-ChR2 driven by a muscle-specific *myo-3* promoter into worms and found that it colocalized well with LMP-1, a worm homologue of the lysosome-associated membrane protein family (Fig 6h). Worms were cultured to the L3 stage at 16°C and then maintained at 25°C for 34 h to induce Aβ expression. A 12-6-12-4-12 light stimulation (alternate light and dark treatment, with the first 12 h of light treatment) was employed to activate lyso-ChR2. The thioflavin S staining showed that the number of Aβ deposits in the CL4176 strain expressing lyso-ChR2 and fedding with all-trans retinal (ATR, a necessary co-factor for ChR2 function that *C*. *elegans* cannot produce) was significantly reduced after light stimulation (Fig 6i and 6j). In contrast, the application of blue light alone (Fig 6j), ATR alone (S9 Fig), or a combination of light and ATR (S9 Fig) did not elicit any significant changes in the amount of Aβ deposits. By observing the number of paralyzed worms with Aβ-induction time, we found that the number of paralyzed worms in lyso-ChR2-expressing CL4176 strain with ATR decreased significantly under light stimulation at the same Aβ-induction time (Fig 6k and S1 Movie). These results implied that lyso-ChR2 activation can alleviate the pathology caused by Aβ accumulation.

## Discussion

Optogenetics has revolutionized the neuroscience field by enabling neuronal activity in selected neurons and brain regions to be activated or inhibited with light, thereby determining their functions in neural circuits and behavior [41]. Recently, optogenetics has been applied to regulate the function and distribution of intracellular organelles [8], but few studies have been performed on lysosomes. Our new tools, lyso-NpHR3.0, lyso-ArchT, and lyso-ChR2, now allow kinetic control of the lysosomal membrane potential, pH, hydrolase activity, Ca^2+^ dynamics, and degradative ability. In particular, lyso-ChR2 activation can induce autophagy and alleviate the phenotype of AD in cellular and *C*. *elegans* models (S10 Fig). These findings suggest that maintaining lysosomal ion homeostasis is crucial for optimal lysosomal function, and targeted modulation of lysosomal ion homeostasis holds promise as a viable therapeutic approach for the management of lysosomal-associated disorders.

Our study provides researchers with versatile tools for dynamically manipulating lysosomal physiology and function to address their specific research objectives. Users can select the most appropriate lysosomal optogenetic tool based on their individual requirements. For instance, lyso-NpHR3.0 is suitable for elevating lysosomal pH, whereas both lyso-ArchT and lyso-ChR2 can be used to decrease lysosomal pH, with lyso-ArchT being particularly effective in achieving rapid acidification. For modulating lysosomal degradation, we recommend the use of lyso-ArchT/ChR2 and lyso-NpHR3.0 to enhance or diminish it, respectively. Lyso-ArchT stands out for promoting lysosomal Ca^2+^ release, while lyso-ChR2 exhibits a unique capability in inducing autophagy. It is essential to emphasize that when utilizing these tools to investigate specific aspects of lysosomal physiology, researchers should be cognizant of the multifaceted effects and consider all dimensions for a comprehensive understanding.

In previous studies, Arch3 and CRY2 were successfully relocated to lysosomes by constructing fusion proteins with the lysosomal membrane proteins CD63 [27] and LAMP [42], respectively. In addition to the essential optogenetic elements, these strategies introduce additional lysosomal membrane proteins CD63 and LAMP, which may potentially impact lysosomal physiology or function. Despite Arch3 disrupting the functionality of CD63 when inserted in its middle region, there remains a possibility that the modified CD63 could still interact with other proteins, potentially introducing non-optogenetic effects. Additionally, an extra transmembrane helix βHK was incorporated by the authors to preserve transmembrane topology, which complicates the construction of plasmids and its impact on lysosomes. Here, we employed a new approach to achieve lysosomal targeting by inserting lysosomal targeting signals EREPLL or GYEVM. The strategy we used greatly reduces the introduction of unnecessary proteins to avoid the impact of overexpression of these proteins on lysosomal function. Furthermore, our constructs have smaller encoding sequences, facilitating easier packaging into AAV vectors for animal experiments. It should be noted that despite the residue of ChR2 on the PM, lysosomal-localized ChR2 is sufficient to cause changes in the physiology and function of lysosomes, while PM-localized ChR2 has no or little effect.

When exploring the lysosomal optogenetic regulation of lysosomal physiology, we found that photoactivation of lyso-ChR2 decreased lysosomal pH (Fig 2g and 2h). ChR2 is a cation-selective channel that is permeable to protons [10]. We expected that activation of lyso-ChR2 would lead to H^+^ efflux from the lysosomal lumen and thus increase lysosomal pH, but the opposite was found. Inhibition of V-ATPase activity with Baf A1 revealed that lyso-ChR2 decreased lysosomal pH by activating V-ATPase (Fig 2i and 2j). Previous studies have shown that activation of TPC channels leads to lysosomal membrane depolarization, which in turn potentiates H^+^ pumping by V-ATPase [18]. Lyso-ChR2 activation also depolarizes the lysosomal membrane potential (Fig 1d), and it is possible that lyso-ChR2 activates V-ATPase through a similar mechanism.

Another unexpected result was the effect of lyso-ChR2 activation on lysosomal Ca^2+^ content. Lysosomes are the second largest Ca^2+^ store inside cells. The Ca^2+^ concentration in the lysosomal lumen is approximately 0.5 mM [43], which is hundreds to thousands of times that of the cytoplasm. Since ChR2 permeates calcium [10], activation of lyso-ChR2 can mediate the release of lysosomal Ca^2+^. However, we detected an increase in lysosomal Ca^2+^ content upon lyso-ChR2 activation (Fig 4g and 4h). In this study, we have not experimentally elucidated the underlying mechanism of this phenomenon. The lysosomal Ca^2+^ content is influenced by various aspects of lysosomal physiology. Therefore, we hypothesize that lyso-ChR2 may indirectly lead to the increase of Ca^2+^ level in lysosomes by influencing lysosomal physiology. Two potential mechanisms are likely involved in this process. Firstly, our data showed that activation of lyso-ChR2 leads to a reduction in lysosomal pH (Fig 2h). The addition of bafilomycin A1, a V-ATPase inhibitor, effectively prevents this pH reduction (Fig 2j), suggesting that lyso-ChR2 reduces lysosomal pH by activating V-ATPase. The H^+^ gradient across the lysosomal membrane has long been recognized as a crucial driving force for lysosomal Ca^2+^ uptake [43,44]. A recent study identified TMEM165 as the protein responsible for mediating this pH-dependent lysosomal Ca^2+^ uptake [45]. It can transport Ca^2+^ into lysosomes against their concentration gradient with the help of low lysosomal pH. Therefore, we propose that the lyso-ChR2-induced reduction in lysosomal pH could enhance Ca^2+^ uptake, potentially through TMEM165-mediated mechanisms, thereby contributing to an elevation in lysosomal Ca^2+^ content. Secondly, our data showed that lyso-ChR2 activation results in a depolarization of lysosomal membrane potential (Fig 1d). This depolarization could reduce the activity of the hyperpolarization-activated Ca^2+^ release channel TRPML1 [46], resulting in a decrease in lysosomal Ca^2+^ release. The combination effect of increased Ca^2+^ uptake and reduced Ca^2+^ release might surpass the Ca^2+^ efflux through the lyso-ChR2 channel, thereby leading to the observed increase in lysosomal Ca^2+^ content presented in Fig 4g and 4h. Notably, PM-localized ChR2 also caused an increase in lysosomal Ca^2+^ content, but not as much as lyso-ChR2 (Fig 4g and 4h), and the mechanism remains unclear.

In addition to regulating lysosomal pH and lysosomal calcium content, we found that activation of lyso-ChR2 also promotes autophagy in an mTOR-dependent manner. mTOR is a master regulator of autophagy. Activated mTORC1 anchors to lysosomes and inhibits multiple steps of the autophagy process, including induction, nucleation, phagophore elongation, and autophagosome maturation [47–51]. Moreover, mTORC1 inhibits the nuclear translocation of TFEB [52,53], a key transcription factor for autophagy and lysosomal biogenesis [34,54]. We found that photoactivation of lyso-ChR2 inhibited mTOR, thereby releasing its inhibitory effect on autophagy and promoting TFEB nuclear import (Fig 5h–5m). However, the mechanism by which lyso-ChR2 inhibits mTOR remains unclear. Previous studies have revealed a connection between lysosomal calcium and mTOR activity. For instance, 2 studies reported that TRPML1-mediated release of lysosomal calcium activates mTORC1 by promoting the binding of calmodulin (CaM) with mTOR and facilitating the recruitment of mTOR onto lysosomes [55,56]. We found that activation of lyso-ChR2 increases lysosomal calcium content (Fig 4g and 4h), possibly due to a reduction in lysosomal calcium release. Therefore, we propose that activation of lyso-ChR2 induces depolarization of the lysosomal membrane potential (as shown in Fig 1d), leading to decreased TRPML1 activity due to its hyperpolarization-activated nature [46], subsequently resulting in diminished lysosomal calcium release. This decrease in calcium release leads to reduced CaM activity and consequently diminishes the recruitment of mTOR onto lysosomes, which in turn promotes autophagy.

Autophagy is a major process by which cells clear damaged organelles and protein aggregates. Conventional methods of regulating autophagy include pharmacological inducers like the mTOR inhibitor rapamycin [57], genetic interventions such as overexpressing the key autophagy protein Beclin1 [58], and stress inducers like starvation. While effective in inducing autophagy, these methods have been limited by a lack of spatial precision and irreversibility. Our optogenetics-based approach effectively addresses these issues. By employing cell-specific promoters, autophagy can be selectively induced in distinct cell types or tissues, thus enhancing spatial resolution. Additionally, refining the illuminated region can also improve the spatial accuracy. Importantly, our approach allows for the reversible induction of autophagy by merely ceasing the light exposure. Finally, the levels of autophagy can be precisely modulated by adjusting light stimulation parameters.

Dysfunction of autophagy has been linked to a variety of neurodegenerative diseases in which misfolded proteins or dysfunctional mitochondria accumulate [59]. Here, we showed that activating autophagy by photoactivation of lyso-ChR2 can clear Aβ and alleviate Aβ-induced pathology in cellular and *C*. *elegans* models of AD, suggesting that modulating lysosomal ion balance to restore autophagy may be an effective therapeutic approach for AD and possibly other neurodegenerative diseases. Validation of the role of lyso-ChR2 in other disease models will be necessary. Our optogenetics-based method for inducing autophagy may provide new ideas for the treatment of AD and potentially other neurodegenerative diseases.

Imbalances in lysosomal ion homeostasis are also cytopathological hallmarks of various diseases. As key regulators of lysosomal ion balance, lysosomal ion channels have been found to be closely related to these diseases and are considered valuable targets for drug development [20]. Lysosomal optogenetics provides a convenient way to manipulate lysosomal ion balance and autophagy and a powerful tool for the research and drug development of lysosome-related diseases. We noted that activating one type of lysosomal optogenetic protein often leads to a cascade of changes in lysosomal physiology and function, which is a disadvantage for the precise manipulation of lysosomes. The reason for this phenomenon is that different physiological parameters of lysosomes are interconnected and affect each other. Similar phenomena have also been found in previous studies on lysosomal ion channels. For example, the activity of the lysosomal potassium channel TMEM175 can also affect lysosomal membrane potential and autophagy [30,60], while the lysosomal chloride channel CLN7 regulates lysosomal calcium signaling and lysosomal pH [25]. These findings also provide further evidence of the dramatic impact of ion homeostasis on lysosomal function. New optogenetic actuators with unique properties are constantly being developed. Applying these new actuators to lysosomes, combined with the control of endogenous lysosomal ion channels and other lysosomal targets, is expected to achieve more precise manipulation of lysosomes in the future. Additionally, our developed lysosome-targeted optogenetic tools, particularly ChR2, still show some residual presence on the plasma membrane (Fig 1b). Enhancing their lysosomal localization is a crucial direction for refining lysosomal optogenetics in the future.

## Materials and methods

### Cell culture

Cells were cultured at 37°C in a humidified CO_2_ (5%) incubator. HEK293T and HeLa cells were maintained in Dulbecco’s Modified Eagle’s Medium (DMEM; Biological Industries) supplemented with 10% fetal bovine serum (FBS; Biological Industries), 1× glutagro (Corning), and 1× penicillin/streptomycin (Biosharp). SH-SY5Y cells were maintained in DMEM/F12 supplemented with 20% FBS, 1× glutagro, and 1× penicillin/streptomycin.

### *Caenorhabditis elegans* strain

The transgenic *C*. *elegans* strain (CL4176) was used in all animal experiments (Fig 6). Worms were propagated at 16°C on nematode growth medium (NGM) (1 mM CaCl_2_, 1 mM MgSO_4_, 5 μg/ml cholesterol, 250 μM KH_2_PO_4_ (pH 6), 17 g/l agar, 3 g/l NaCl, 7.5 g/l casein) plates and seeded with *E*. *coli* OP50. The expression of muscle-specific Aβ_1–42_ in transgenic CL4176 worms was induced by increasing the temperature from 16 to 25°C.

### Cloning strategy and transfections

To generate lyso-ChR2, full-length cDNA of ChR2 from AAV-ChR2-mCherry (gift from Dr. Tian Xue, University of Science and Technology of China) was amplified by PCR. Next, the lysosomal sorting signal GYEVM was amplified and inserted into the C-terminus of ChR2. Finally, the recombinant fragment was subcloned into the *Nhe*I and *Sac*I sites of the pEGFP-N1 or mCherry2-N1 vector. pEGFP-N1-ChR2-GYEQF was generated in the same way. ChR2-EREPLL, ChR2-2×EREPLL, and ChR2-PQLC2-C were subcloned into the *Nhe*I and *Hin*dIII sites of the pEGFP-N1 or mCherry2-N1 vector to create mCherry2-N1-ChR2-EREPLL, pEGFP-N1-ChR2-2×EREPLL, and pEGFP-N1-ChR2-PQLC2-C, respectively. To generate lyso-NpHR3.0 and lyso-ArchT, full-length cDNAs of NpHR3.0/ArchT from AAV-eNpHR3.0/CamKII-ArchT-GFP (gift from Dr. Tian Xue, University of Science and Technology of China) with the lysosomal sorting signal EREPLL inserted into the C-terminus were subcloned into the *Sac*I and *Hin*dIII sites of the pEGFP-N1 or mCherry2-N1 vector. To create lyso-ArchT-GCaMP6m, full-length cDNA of GCaMP6m from CMV-GCamP6m-C1 (gift from Dr. Guoqiang Bi, University of Science and Technology of China) was inserted at the C-terminus of ArchT via the *Hin*dIII and *Eco*RI sites. Transient expression of DNA constructs was performed with Lipofectamine 2000 (Invitrogen) according to the manufacturer’s protocol.

For lentiviral expression constructs, the cDNAs of ChR2-GYEVM, NpHR3.0-EREPLL, and ArchT-EREPLL were PCR-amplified and subcloned into *Eco*RI and *Bam*HI sites of the pSin-EF2-puro vector. Lentivirus was produced according to published protocols. Briefly, the lentiviral expression constructs were cotransfected into HEK293T cells with helper plasmids psPAX2 and pMD2.G via LentiFit (Hanbio). Lentiviral particles were harvested from the culture medium at 48 h and 72 h after transfection. Supernatants were filtered and subsequently concentrated 100-fold using ultracentrifugation (40,000 rpm, 2 h). Aliquots of the lentivirus were frozen and stored at −80°C.

To generate lyso-ChR2 with or without GFP tag for expression in worm muscle, a 2 kb promoter region of muscle-specific gene *myo-3* (P_*myo-3*_) and codon-optimized cDNA of ChR2 with lysosomal targeting signal GYEVM were recombined with *Sph*I/*Age*I or *Sph*I/*Eco*RI-linearized pPD117.01 vector (Addgene #1587) using a standard homologous recombination reaction. For RFP-tagged LMP-1, the 2 kb promoter region of *myo-3* (P_*myo-3*_) and the cDNA of lmp-1 were recombined with *Pst*I/*Age*I-lineared pPD95.67 vector (Addgene #1490) in which the original reporter GFP was replaced by RFP. Plasmids were injected into wild-type or mutant animals at a total concentration of 20 ng/μl.

### Electrophysiology

Electrophysiological recordings were carried out at room temperature using a Multiclamp 700B (Molecular Device) for HEK293T cells. A Digidata 1500B (Molecular Devices) under the control of pClamp software (Molecular Device) was used for data acquisition. Clampfit (Molecular Devices) and OriginPro (OriginLab) software were used for data analysis. Whole-endolysosome recordings were performed with enlarged endolysosomes treated with vacuolin-1 (1 μM, overnight) according to published protocols. The bath solution used for endolysosomal recordings contained (in mM): 140 K-gluconate, 4 NaCl, 2 MgCl_2_, 0.39 CaCl_2_, 1 EGTA, 10 HEPES, pH 7.2 by KOH. The pipette solution contained (in mM): 145 NaCl, 5 KCl, 1 MgCl_2_, 2 CaCl_2_, 10 HEPES, 10 MES, and 10 glucose, pH 4.6 by HCl.

### Light stimulation

An LED light stimulation system (Hangzhou Braintech Technology) was used for long-term stimulation. For lyso-ChR2 stimulation, blue light with a wavelength of 470 nm (10 Hz, 50 ms, 6 h, 5.6 mW/cm^2^) was applied. The lyso-ChR2 in Fig 6a–6g, S8a and S8c Fig was stimulated for 12 h under the same conditions. For lyso-NpHR3.0/ArchT stimulation (Fig 3a, 3c and 3e, S4a, S5c and S5d Figs), yellow light with a wavelength of 589 nm (5 Hz, 80 ms, 6 h, 2.15 mW/cm^2^) was applied.

### Live cell imaging

Images were taken using a confocal system consisting of an Eclipse Ti inverted microscope (Nikon), a CSU-X1 Spinning Disk unit (Yokogawa), a DU-897U EMCCD camera (Andor), a laser-controlling module (Andor), and iQ3 imaging software (Andor) with a 100× oil immersion lens. For protein localization (Fig 1b and S2 Fig), HEK293T cells were transfected for at least 6 h and then trypsinized to seed into chambers with thin glass bottoms coated with poly-L-lysine.

For the autophagosome-lysosome fusion assay, cells were transfected with mCherry-GFP-LC3 and plated onto poly-L-lysine-coated confocal dishes. For the EGFP-2× FYVE assay, cells were transfected with EGFP-2× FYVE and plated onto poly-L-lysine-coated confocal dishes. For the nuclear translocation assay, cells were transfected with TFEB-mCherry and plated onto poly-L-lysine-coated confocal dishes. All experiments were performed 24 h after transfection, and the cells were stimulated by LED light for 6 h. Imaging was performed with a Plan-APOCHROMAT 100×/1.4 Oil DIC oil immersion objective on an LSM980 confocal microscope (Zeiss).

### Lysosome pH imaging

pHluorin imaging was carried out in HEK293T cells cotransfected with lyso-NpHR3.0/lyso-ArchT and pCMV-lyso-pHluorin (Addgene, #70113). All experiments were performed 24 h after transfection. lyso-NpHR3.0/lyso-ArchT was stimulated at 594 nm, while pHluorin fluorescence was excited at 488 nm by laser.

pHrodo dextran was used to monitor lysosomal pH changes in lyso-ChR2-expressing cells (Fig 2g–2l). Cells were incubated with pHrodo dextran (10 μg/ml; Thermo Fisher Scientific) for 6 h at 37°C and then cultured in medium without pHrodo dextran for an additional 1 h. Bafilomycin A1 (1 μM, MCE) was used to inhibit V-ATPase (Fig 2c, 2d, 2i and 2j). Imaging was performed with a Plan-APOCHROMAT 100×/1.4 Oil DIC oil immersion objective on an LSM980 confocal microscope (Zeiss).

### Lysosome calcium imaging

OG-BAPTA 5N was used for lysosomal calcium imaging of lyso-NpHR3.0 (Fig 4a–4d). HEK293T cells were transfected with lyso-NpHR3.0 and plated onto poly-L-lysine-coated confocal dishes. Cells were incubated with OG-BAPTA 5N (20 μM; Thermo Fisher Scientific) for 12 h at 37°C and then cultured in medium without OG-BAPTA 5N for another 2 h. Imaging was performed with a Plan-APOCHROMAT 100×/1.4 Oil DIC oil immersion objective on an LSM980 confocal microscope (Zeiss).

For calcium imaging of lyso-ArchT, GCaMP6m, a genetically encoded Ca^2+^ sensor, was employed. HEK293T cells were transfected with lyso-ArchT-GCaMP6m and plated onto poly-L-lysine-coated confocal dishes. All experiments were performed 24 h after transfection. Lysosomal Ca^2+^ release was measured under a zero-Ca^2+^ external solution, which contained 145 mM NaCl, 5 mM KCl, 3 mM MgCl_2_, 10 mM glucose, 1 mM EGTA, and 20 mM HEPES (pH 7.4). Lyso-ArchT was activated at 594 nm, and GCaMP6m fluorescence was excited at 488 nm by laser.

For calcium imaging of lyso-ChR2, cells were plated onto poly-L-lysine-coated coverslips and stimulated by light for 6 h. Furo-2 AM (Beyotime) was used to measure the calcium ions released from lysosomes according to the manufacturer’s protocol. In brief, 2 μM Fura-2 AM and 0.02% pluronic F-127 were incubated for 30 min at 37°C in isotonic solution containing 135 mM NaCl, 5.4 mM KCl, 1.8 mM CaCl_2_, 0.9 mM MgCl_2_, 10 mM glucose, and 10 mM HEPES (pH 7.4 by NaOH). The same solution but without CaCl_2_ was used as the imaging buffer. Cells were then washed twice with imaging buffer and transferred into the imaging chamber. GPN (600 μM) was applied to the cells to induce Ca^2+^ release from lysosomes. Calcium transients were captured using a calcium imaging system consisting of a DG-5 wavelength switcher (Sutter Instrument), an ORCA-Flash4.0 LT+ complementary metal–oxide–semiconductor (CMOS) camera (Hamamatsu), and a Ti2 microscope (Nikon). Data were collected and analyzed using MetaFluor software (Molecular Devices). Ratiometric measurements were performed by switching the excitation wavelength from 340 to 380 nm and quantifying emission at 510 ± 40 nm.

### Magic Red cathepsin activity detection assay

Cells grown on poly-L-lysine-coated confocal dishes were stained with Magic Red Cathepsin-B or Cathepsin-L Assay Kit (Immuno Chemistry Technologies) for 30 min at 37°C. After staining, the cells were washed twice in PBS and incubated in cell culture medium. Imaging was performed with a Plan-APOCHROMAT 100×/1.4 Oil DIC oil immersion objective on an LSM980 confocal microscope (Zeiss).

### DQ-Red BSA assay

For degradation ability analysis, cells were loaded with DQ-Red BSA (20 μg/ml, Invitrogen) for 1 h at 37°C and then incubated with fresh culture medium without DQ-Red BSA for another 1 h. After incubation, the cells were washed with PBS and fixed with 4% paraformaldehyde (PFA) immediately. Imaging was performed with a Plan-APOCHROMAT 100×/1.4 Oil DIC oil immersion objective on an LSM980 confocal microscope (Zeiss).

### Western blotting and antibodies

HEK293T cells were lysed with NP-40 lysis buffer supplemented with complete protease inhibitor cocktail tablets (Thermo Fisher Scientific). After centrifugation at 18,500 ×g for 10 min, the supernatants were collected. The total protein concentration was determined using a BCA Protein Assay Kit (Beyotime). Total cell lysates were mixed with 5× SDS-loading buffer and boiled at 100°C for 5 min. Protein samples (20 μg) were loaded onto a 15% SDS-polyacrylamide electrophoresis (SDS-PAGE) gel and separated at a constant voltage of 100 V for 2 h. Then, the samples were transferred onto polyvinylidene difluoride (PVDF) membranes using a transblot electrophoretic transfer system (Cavoy). Transblotting was performed at a constant current of 150 mA for 1 h at 4°C. Membranes were blocked for 2 h in Tris-buffered saline (TBS) buffer with 5% skimmed milk and 0.1% Tween-20. The antibodies used included LC3B (ab51520, Abcam), mTOR (7C10) (2983, Cell Signaling), phospho-mTOR (Ser2448) (67778-1-Ig, Proteintech), Aβ (sc-28365, Santa Cruz), GAPDH (60004-1-Ig, Proteintech), peroxidase-conjugated immunopure goat anti-rabbit IgG (H+L) (SA00001-2, Proteintech), and anti-mouse IgG (H+L) (SA00001-1, Proteintech). Enhanced chemiluminescence horseradish peroxidase was used to visualize the protein bands.

### Immunofluorescence and co-localization analysis

Immunofluorescence and co-localization analysis were used in Fig 5j and 5k. Cells were plated onto poly-L-lysine-coated confocal dishes. After treatments, cells were fixed with 4% paraformaldehyde/phosphate-buffered saline (PBS) at room temperature for 10 min and washed/permeabilized with 1× PBST solution (1× PBS and 0.1% Triton X-100) for another 15 min. Then, the cells were blocked with blocking solution (1× PBS with 1% BSA) at room temperature for 30 min. Staining was performed with the indicated primary antibodies in blocking solution (1:200 dilution) at 4°C overnight. The cells were stained with cross-adsorbed secondary fluorescent antibodies (1:100 in blocking solution). For the merged images, Elab Fluor 594 (E-AB-1059, Elabscience, for anti-LAMP2 IFs) and Elab Fluor 488 (E-AB-1055, Elabscience, for anti-mTOR IFs) are shown in magenta and cyan, respectively. Imaging was performed with a 100× oil immersion objective on Olympus confocal microscope. The antibodies used included LAMP2 (66301-1-Ig, Proteintech), mTOR (7C10) (2983, Cell Signaling).

To quantify the co-localization of mTOR with the lysosomal marker LAMP2, the Fiji software was used. Forty individual cells from independent fields were selected for the analysis. The Coloc2 plugin was used to calculate the Pearson’s correlation coefficient.

### shRNA knockdown

The pLKO.1 vector (gift from Dr. Dan Liu, University of Science and Technology of China) was used for shRNA knockdown. The targeted sequence for h*Atg7* was GCCTGCTGAGGAGCTCTCCAT. To generate shRNA-encoding plasmids, oligo pairs [for h *Atg7*, CCGGGCCTGCTGAGGAGCTCTCCATCTCGAGATGGAGAGCTCCTCAGCAGGCTTTTTG (forward), AATTCAAAAAGCCTGCTGAGGAGCTCTCCATCTCGAGATGGAGAGCTCCTCAGCAGGC (reverse)] were annealed and ligated into the *Age*I and *Eco*RI sites of the pLKO.1 vector. Scramble shRNA was a gift from Dr. Dan Liu. The procedures for the production of lentiviruses and transfection and selection of puromycin-resistant cells were the same as mentioned above.

### Enzyme-linked immunosorbent assay

Cells were transfected with APPswe using a jetPRIME kit (114–15, polyplus) for 12 h. Then, sh*Atg7* or scramble control shRNA was applied to cells for another 12 h and plated onto poly-L-lysine-coated 24-well cell culture plates. Cells were then stimulated by LED light for 12 h. After treatment, the medium was collected, and Aβ_1–42_ levels were measured by sandwich ELISA.

### Amyloid-beta peptide clearance assay

Cells grown on poly-L-lysine-coated confocal dishes were loaded with Fluorochrome-tagged Aβ_1–42_ HiLyte Fluor 555 (1 μg/ml, ANASPEC) for 12 h. Cells were then washed twice with PBS and incubated with cell culture medium before light stimulation. Imaging was performed with a Plan-APOCHROMAT 100×/1.4 Oil DIC oil immersion objective on an LSM980 confocal microscope (Zeiss).

### Fluorescence staining of Aβ deposits

After Aβ induction and light stimulation, worms were collected and washed with M9 (3 g/l KH_2_PO_4_, 6 g/l Na_2_HPO_4_, 5 g/l NaCl, 1 μM MgSO_4_) buffer 3 times. Then, worms were fixed in 4% paraformaldehyde/PBS at 4°C for 24 h and permeabilized in a solution of 5% mercaptoethanol, 1% Triton-X, and 25 mM Tris-HCl (pH 7.4) at 37°C for another 24 h. After washing 3 times with PBS-T (PBS plus 0.1% Triton X-100), the worms were stained with 0.125% thioflavin S (MCE) in 50% ethanol for 2 min and then destained with 50% ethanol 3 times (2 min each). Stained worms were resuspended in 100 μl PBS, and images were taken by fluorescence microscopy (Zeiss, LSM 980).

### Worm paralysis assay

Temperature-sensitive Aβ strains (CL4176) were maintained at 16°C. The lyso-ChR2 plasmids, along with a fluorescence marker pCFJ90 (Addgene #19327), were injected into CL4176 worms, and then mCherry-positive progenies were picked out for subsequent experiments. Worms were induced to express human Aβ until they were at the L3 stage by upshifting the temperature from 16°C to 25°C after being transferred to NGM plates, with or without ATR as indicated. Concurrently, the worms were exposed to a 12-6-12-4-12 h lighting (470 nm, 10 Hz and 50-ms stimulus intervals, 5.6 mW/cm^2^) regime in which light was ON for 12 h, followed by light OFF for 6 h and light ON for another 12 h. The number of worms was counted after the 4 h light OFF, and the light was kept on for 12 h during counting. Worms were scored every 2 h for paralysis until worms in the control group were all paralyzed. When worms did not move or only moved their heads to gently touch their own tails, paralysis was considered. Three independent experiments were performed for paralysis analysis.

### MTT assay

Cells were plated onto poly-L-lysine-coated 24-well cell culture plates and then stimulated by LED light for 6 h. Cell viability was detected using the MTT Cell Proliferation Kit (E606334, Sangon Biotech).

### Quantitative real-time PCR (qRT-PCR)

Total RNA was extracted using Trizol extraction kits (Vazyme) according to the manufacturer’s protocol. Reverse transcription was performed using Hiscript III Reverse Transcriptase (Vazyme). Approximately 1 μg of the total RNA was used as the template for reverse transcription. The following primers were used: 5′-CATCGGGACTATCGTGTGGG-3′ (forward) and 5′-GCGCCATAGCACAATCCAAG-3′ (reverse) for ChR2; 5′-TGGGGACTTGTGTCCAAACC-3′ (forward) and 5′-GCCAAGCTTGTAACCAGGGA-3′ (reverse) for *Atg7* and 5′-ATCATCAGACCACAGTCCATGC-3′ (forward) and 5′-CCAGTGAGCTTCCCGTTCAG-3′ (reverse) for GAPDH. The 2^-ΔΔCt^ method was used to calculate the relative mRNA levels of ChR2 and *Atg7*.

### Data analysis

Images were analyzed using ImageJ software. Statistical analysis was performed using Clampfit (Molecular Devices), SigmaPlot (Systat Software), and OriginPro (OriginLab). For statistical analysis of differences between 2 groups, only Student’s *t* test was used. For group comparison in Fig 6k, one-way analysis of variance (ANOVA) was performed. Values of data are shown as the mean ± SEM, and a significant difference was considered when *p* < 0.05.

## Supporting information

S1 Fig Lysosome-targeted optogenetic actuators are localized on enlarged lysosomal membranes. Colocalization of C-terminally mCherry-tagged lyso-NpHR3.0 (upper, magenta) or lyso-ArchT (middle, magenta) with LysoTracker Green (cyan), and C-terminally EGFP-tagged lyso-ChR2 (lower, cyan) with LysoTracker Red (magenta) in HEK293T cells. HEK293T cells were pretreated with 1 μM vacuolin-1 overnight to enlarge lysosomes. Arrows indicate enlarged lysosomes. Scale bar, 10 μm.
(TIF)

S2 Fig Localizations of ChR2 by different lysosomal targeting strategies. Colocalization of LysoTracker Green/Red with ChR2 carrying lysosomal targeting signals EREPLL (a), 2×EREPLL (b), PQLC2-C (c), or GYEQF (d) at its C-terminus. A magnified image of the white boxed area is shown in the upper right corner of the merged image. Scale bar, 10 μm.
(TIF)

S3 Fig Lysosome-targeted optogenetic actuators stably transfected into HEK293T cells display normal functional properties. (a, f, and k) Relative mRNA levels of NpHR3.0 (a), ArchT (f), or ChR2 (k) in HEK293T cells stably transfected with lyso-NpHR3.0 (a), lyso-ArchT (f), lyso-ChR2 (k), or the corresponding vector (as control). *n* = 6 in each group. (b, g, and l) Lysosomal membrane currents evoked by brief corresponding light stimulation in HEK293T cells stably expressing lyso-NpHR3.0 (b), lyso-ArchT (g), or lyso-ChR2 (l). (c, h, and m) Statistics of light-induced lysosomal membrane currents in b, g, and l. *n* = 8, 5, and 10 for c, h, and m, respectively. (d, i, and n) Lysosomal membrane potentials evoked by brief corresponding light stimulation in HEK293T cells stably expressing lyso-NpHR3.0 (d), lyso-ArchT (i), or lyso-ChR2 (n). (e, j, and o) Statistics of light-induced lysosomal membrane currents in HEK293T cells transiently or stably transfected with lyso-NpHR3.0 (e), lyso-ArchT (j), or lyso-ChR2 (o). The stable expression currents for e, j, and o originate from c, h, and m, respectively, for comparative analysis. Data are shown as mean ± SEM. * *P* < 0.05, ** *P* < 0.01, ns, not significant. The data underlying this figure can be found in S1 Data.
(TIF)

S4 Fig Cell viability of HEK293T cells is not affected by prolonged light stimulation. MTT assay was used to analyze the cell viability of HEK293T cells stably expressing lyso-NpHR3.0 (a), lyso-ArchT (a), vector (a), lyso-ChR2 (b), or ChR2 (b) after yellow (a) or blue (b) light stimulation for 6 h. *n* = 4 for each group. Data are shown as mean ± SEM. ns, not significant. The data underlying this figure can be found in S1 Data.
(TIF)

S5 Fig Lyso-ChR2 activation dynamically regulates autophagy in HEK293T cells. (a and b) Immunoblotting analysis of the protein levels of LC3-I and LC3-II in HeLa (a) or SH-SY5Y (b) cells transfected with lyso-ChR2 or empty vector. (c) Immunoblotting analysis of LC3-I and LC3-II levels in HEK293T cells stably expressing lyso-NpHR3.0, lyso-ArchT, or vector, with or without 6 h yellow light stimulation. (d) The quantified ratio of LC3-II to LC3-I in c. *n* = 4. (e–g) Protein levels of LC3-I and LC3-II in HEK293T cells stably expressing lyso-ChR2 or empty vector under different light stimulation time (e), intensity (f), or pulse width (g) as indicated. Data are shown as mean ± SEM. ns, not significant. The data underlying this figure can be found in S1 Data.
(TIF)

S6 Fig Activation of lyso-ChR2 induces an increase in the number of lysosomes. (a) Representative images of HEK293T cells stably expressing lyso-ChR2 or PM-targeted ChR2 stained with LysoTracker Red, with or without blue light. Scale bar, 10 μm. (b) Statistics of the number of lysosomes per cell in (a). *n* = 51, 46, 43, and 46 for bars from left to right. Data are shown as mean ± SEM. *** *p* < 0.001; ns, not significant. The data underlying this figure can be found in S1 Data.
(TIF)

S7 Fig Colocalization of EGFP-tagged lyso-ChR2 (I197S) (cyan) with LysoTracker Red (magenta) in HEK293T cells. Scale bar, 10 μm.
(TIF)

S8 Fig Knockdown of *ATG7* inhibits lyso-ChR2 activation-induced autophagy. (a) The protein levels of LC3-I and LC3-II in HEK293T cells stably expressing lyso-ChR2 in response to *ATG7* knockdown, with or without 6 h blue light stimulation. (b) Relative *ATG7* mRNA level in lyso-ChR2-expressing HEK293T cells infected with shRNA against *ATG7* (shATG7) or a scramble control. *n* = 3. (c) ELISA analysis of extracellular Aβ concentration in lyso-ChR2-expressing HEK293T cells in response to *ATG7* knockdown, with or without 6 h blue light stimulation. *n* = 4. Data are shown as mean ± SEM. * *P* < 0.05, *** *p* < 0.001. The data underlying this figure can be found in S1 Data.
(TIF)

S9 Fig ATR alone or in combination with blue light does not influence Aβ induction in the CL4176 strain of *C*. *elegans*. (a) Representative images of CL4176 strains stained with thioflavin S, in the presence of ATR alone or ATR with blue light exposure. Arrows indicate Aβ deposits. Scale bar, 10 μm. (b) The number of Aβ deposits in the head region/anterior area of the worms in d. *n* = 21, 23, and 21 worms for bars from left to right. Data are shown as mean ± SEM. ns, not significant. The data underlying this figure can be found in S1 Data.
(TIF)

S10 Fig A model illustrating that photoactivation of lysosome-targeted optogenetic actuators regulate lysosomal physiology and enhance Aβ clearance through the autophagic pathway. The optogenetic actuators are modified with lysosomal targeting signals to target the lysosomal membrane. Photoactivation of lysosome-targeted optogenetic actuators regulates lysosomal membrane potential, pH, Ca^2+^ dynamics, cathepsins activity, and degradation (left). In addition, activation of lysosome-targeted ChR2 (lyso-ChR2) induces autophagy through the mTOR pathway and enhance Aβ clearance through the autophagic pathway (right).
(TIF)

S1 Movie Lyso-ChR2 activation alleviates the paralytic phenotype of Alzheimer’s disease in *C*. *elegans*. CL4716 worms in each group were treated with the same Aβ-induction time and light stimulation. As shown in the movie, only the CL4176 worms expressing lyso-ChR2 and adding ATR could move their body under light stimulation at 46 h of Aβ induction.
(MP4)

S1 Data Numerical data of figures. Excel spreadsheet containing, in separate sheets, the underlying numerical data and statistical analysis for Figs 1d, 2b, 2d, 2f, 2h, 2j, 2l, 3a, 3b, 3c, 3d, 3e, 3f, 4b, 4d, 4f, 4g, 4h, 5b, 5d, 5e, 5g, 5i, 5k, 5m, 6e, 6g, 6j, 6k, S3a, S3b, S3c, S3d, S3e, S3f, S3g, S3h, S3i, S3j, S3k, S3l, S3m, S3n, S3o, S4a, S4b, S5d, S6b, S8b, S8c, and S9b.
(XLSX)

S1 Raw Images Original images for blots and gels. (PDF)

## Abbreviations

AD: Alzheimer’s disease
ATR: all-trans retinal
CMOS: complementary metal–oxide–semiconductor
DMEM: Dulbecco’s Modified Eagle’s Medium
FBS: fetal bovine serum
LSD: lysosomal storage disease
NGM: nematode growth medium
PBS: phosphate-buffered saline
PVDF: polyvinylidene difluoride
qRT-PCR: quantitative real-time PCR
TFEB: transcription factor EB
