# Supplementary figures and images for: Optogenetic manipulation of lysosomal physiology and autophagy-dependent clearance of amyloid beta
Source: PLoS Biol. 2024 Apr 23;22(4):e3002591. doi: 10.1371/journal.pbio.3002591 (PMC11068202; doi:10.1371/journal.pbio.3002591)

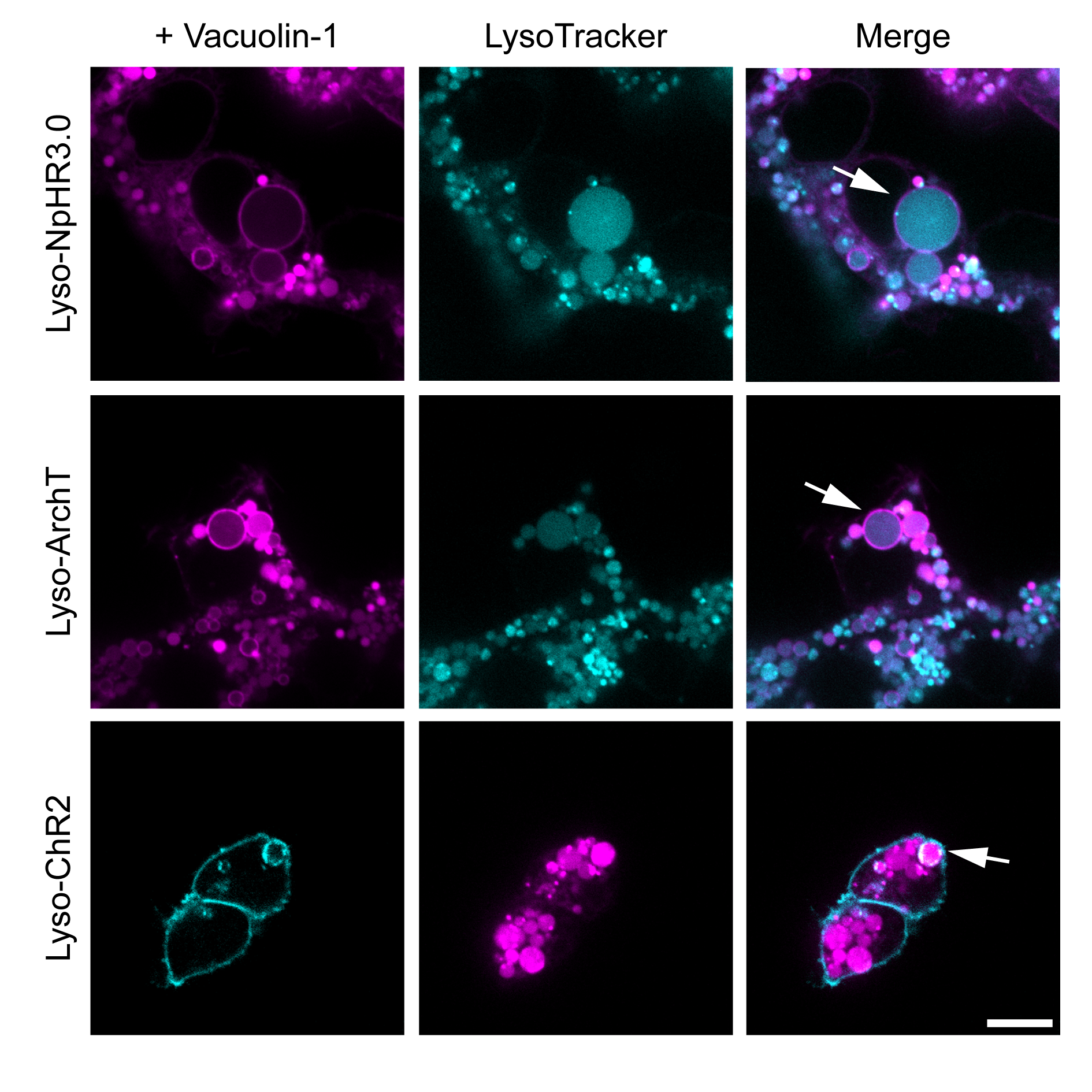

Supplement: S1 Fig — Colocalization of C-terminally mCherry-tagged lyso-NpHR3.0 (upper, magenta) or lyso-ArchT (middle, magenta) with LysoTracker Green (cyan), and C-terminally EGFP-tagged lyso-ChR2 (lower, cyan) with LysoTracker Red (magenta) in HEK293T cells. HEK293T cells were pretreated with 1 μM vacuolin-1 overnight to enlarge lysosomes. Arrows indicate enlarged lysosomes. Scale bar, 10 μm. (TIF) [file pbio.3002591.s001.tif]

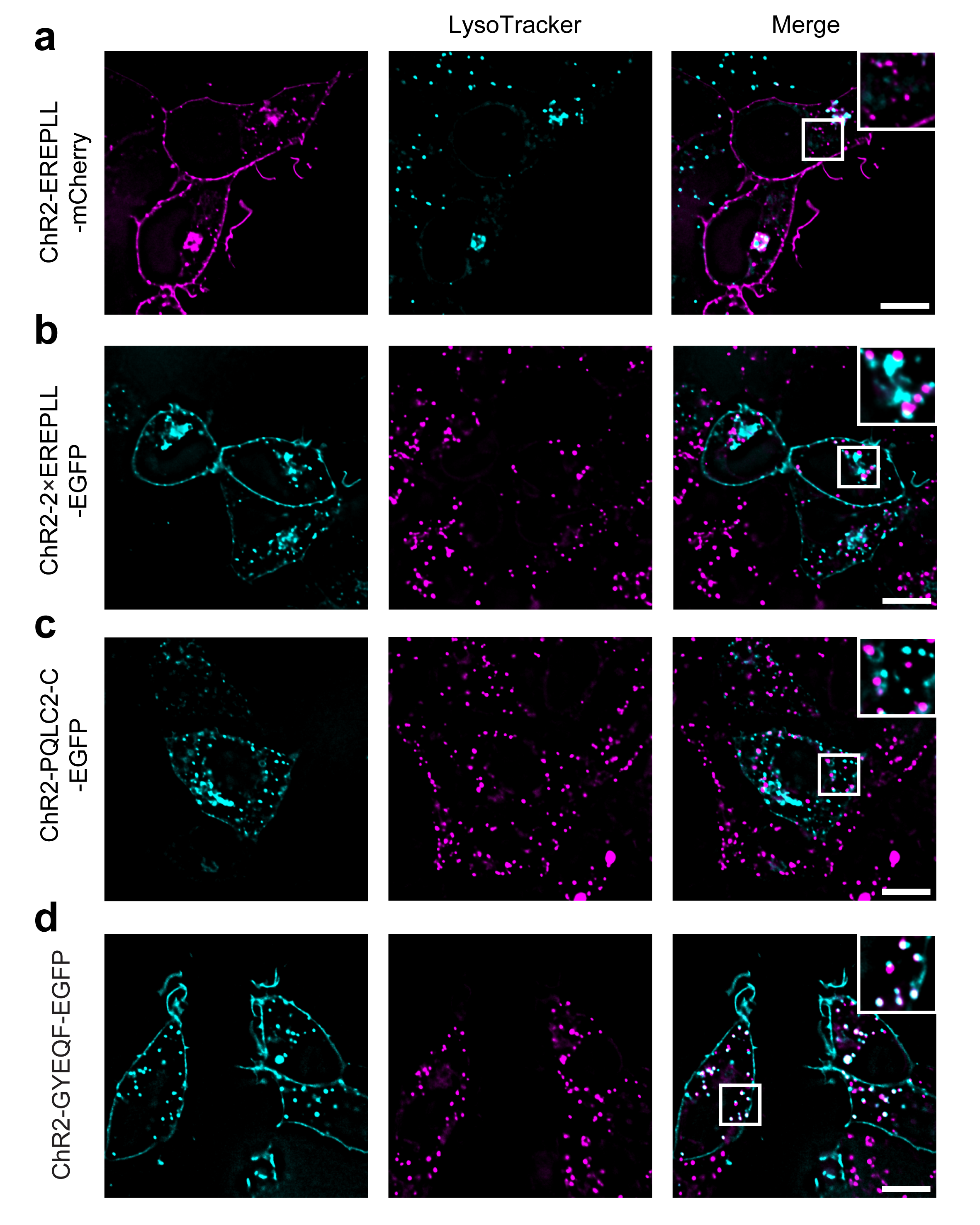

Supplement: S2 Fig — Colocalization of LysoTracker Green/Red with ChR2 carrying lysosomal targeting signals EREPLL (a), 2×EREPLL (b), PQLC2-C (c), or GYEQF (d) at its C-terminus. A magnified image of the white boxed area is shown in the upper right corner of the merged image. Scale bar, 10 μm. (TIF) [file pbio.3002591.s002.tif]

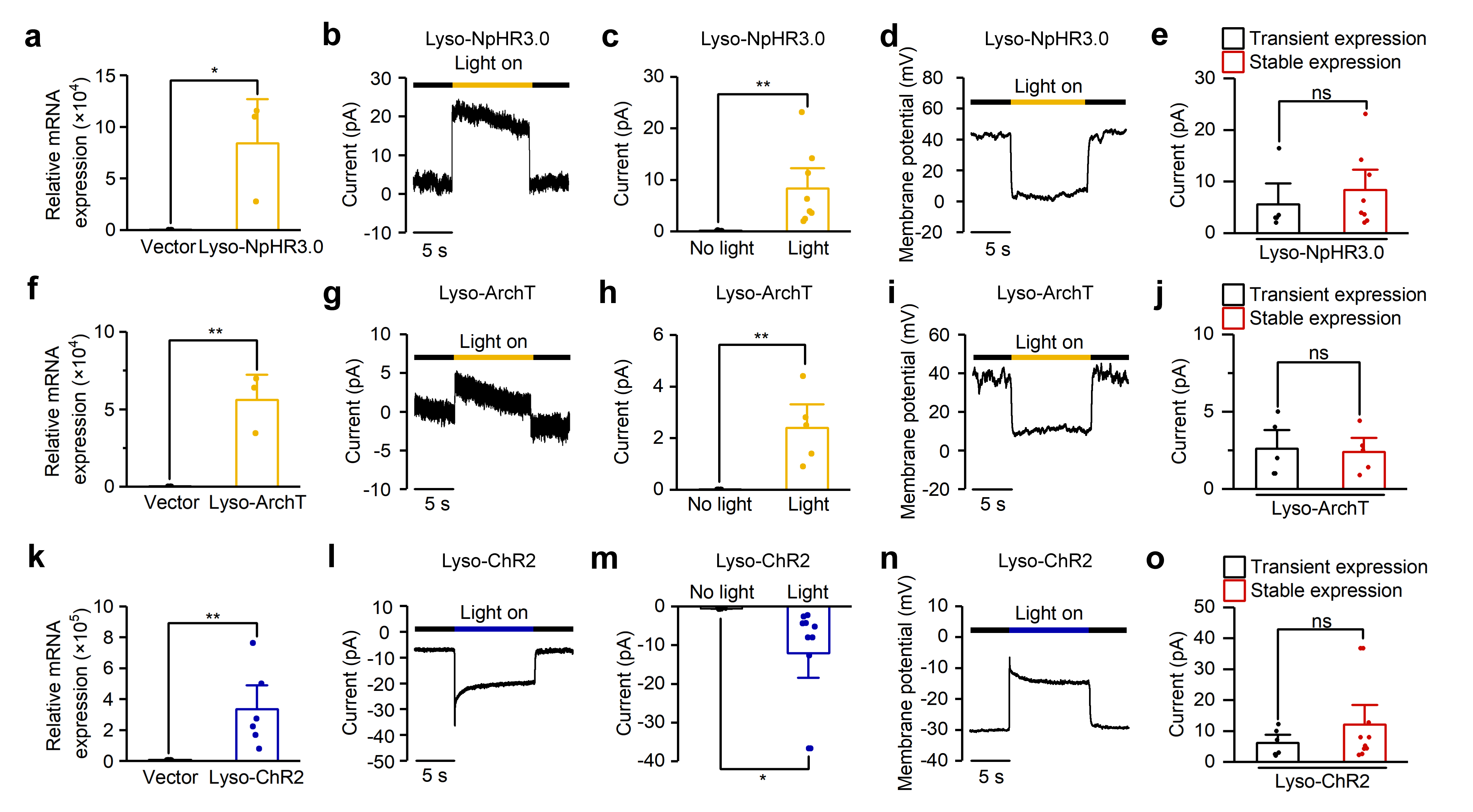

Supplement: S3 Fig — (a, f, and k) Relative mRNA levels of NpHR3.0 (a), ArchT (f), or ChR2 (k) in HEK293T cells stably transfected with lyso-NpHR3.0 (a), lyso-ArchT (f), lyso-ChR2 (k), or the corresponding vector (as control). n = 6 in each group. (b, g, and l) Lysosomal membrane currents evoked by brief corresponding light stimulation in HEK293T cells stably expressing lyso-NpHR3.0 (b), lyso-ArchT (g), or lyso-ChR2 (l). (c, h, and m) Statistics of light-induced lysosomal membrane currents in b, g, and l. n = 8, 5, and 10 for c, h, and m, respectively. (d, i, and n) Lysosomal membrane potentials evoked by brief corresponding light stimulation in HEK293T cells stably expressing lyso-NpHR3.0 (d), lyso-ArchT (i), or lyso-ChR2 (n). (e, j, and o) Statistics of light-induced lysosomal membrane currents in HEK293T cells transiently or stably transfected with lyso-NpHR3.0 (e), lyso-ArchT (j), or lyso-ChR2 (o). The stable expression currents for e, j, and o originate from c, h, and m, respectively, for comparative analysis. Data are shown as mean ± SEM. * P < 0.05, ** P < 0.01, ns, not significant. The data underlying this figure can be found in S1 Data. (TIF) [file pbio.3002591.s003.tif]

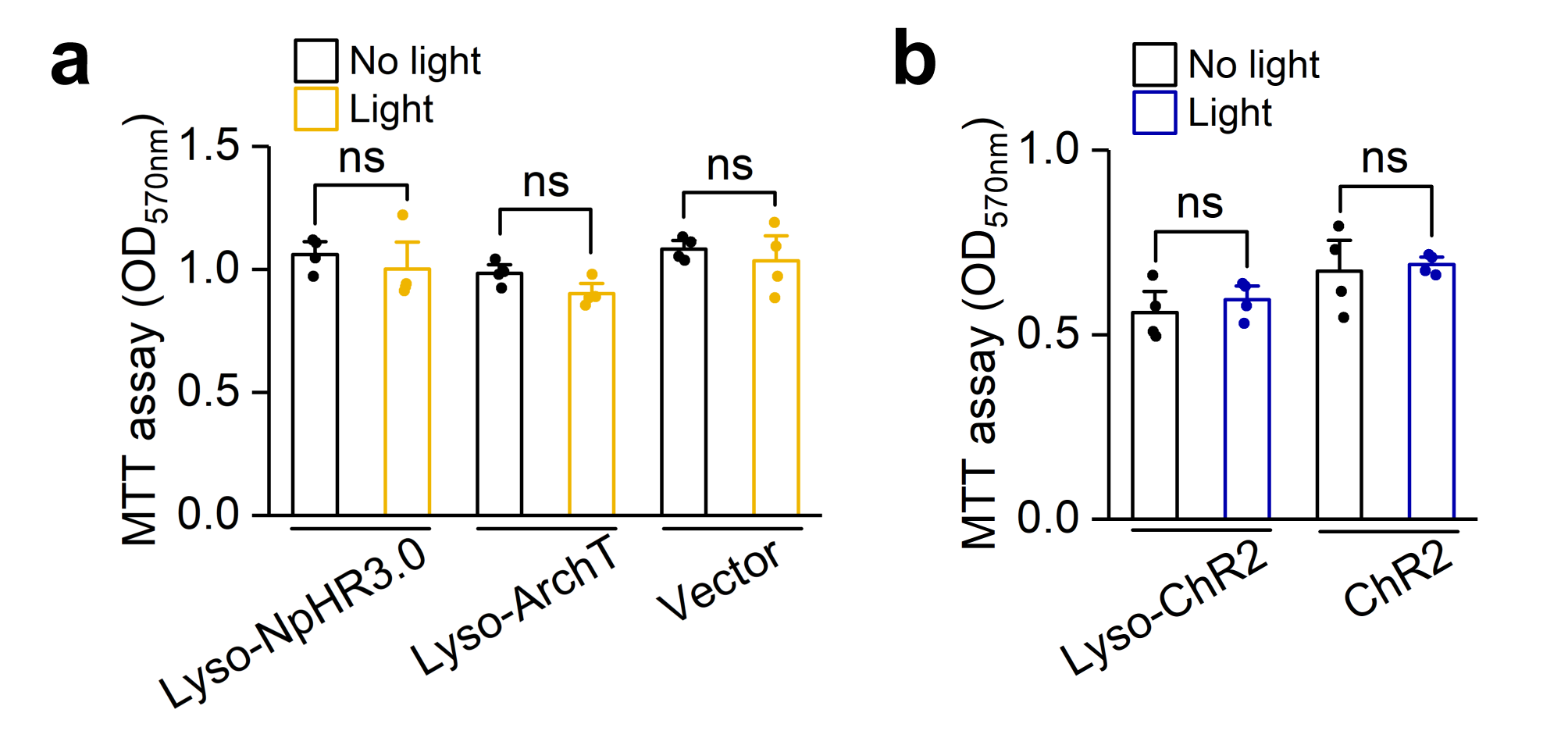

Supplement: S4 Fig — MTT assay was used to analyze the cell viability of HEK293T cells stably expressing lyso-NpHR3.0 (a), lyso-ArchT (a), vector (a), lyso-ChR2 (b), or ChR2 (b) after yellow (a) or blue (b) light stimulation for 6 h. n = 4 for each group. Data are shown as mean ± SEM. ns, not significant. The data underlying this figure can be found in S1 Data. (TIF) [file pbio.3002591.s004.tif]

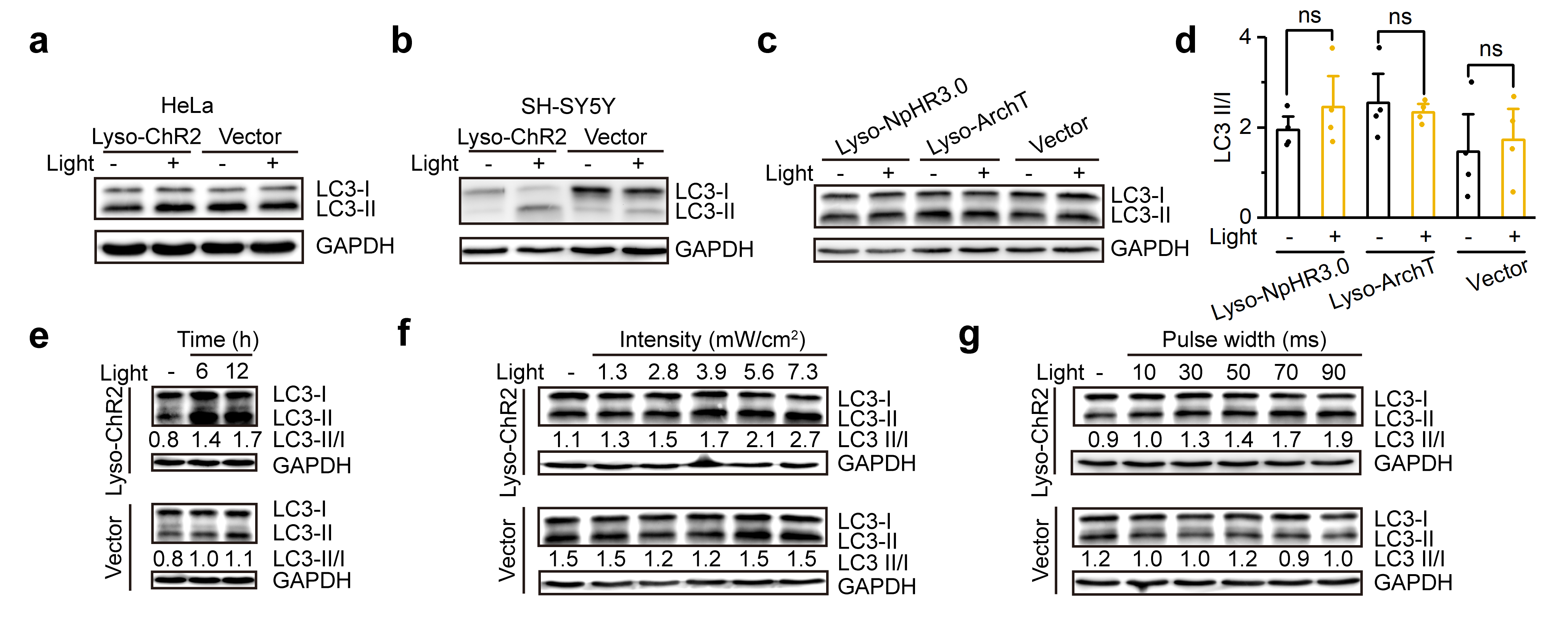

Supplement: S5 Fig — (a and b) Immunoblotting analysis of the protein levels of LC3-I and LC3-II in HeLa (a) or SH-SY5Y (b) cells transfected with lyso-ChR2 or empty vector. (c) Immunoblotting analysis of LC3-I and LC3-II levels in HEK293T cells stably expressing lyso-NpHR3.0, lyso-ArchT, or vector, with or without 6 h yellow light stimulation. (d) The quantified ratio of LC3-II to LC3-I in c. n = 4. (e–g) Protein levels of LC3-I and LC3-II in HEK293T cells stably expressing lyso-ChR2 or empty vector under different light stimulation time (e), intensity (f), or pulse width (g) as indicated. Data are shown as mean ± SEM. ns, not significant. The data underlying this figure can be found in S1 Data. (TIF) [file pbio.3002591.s005.tif]

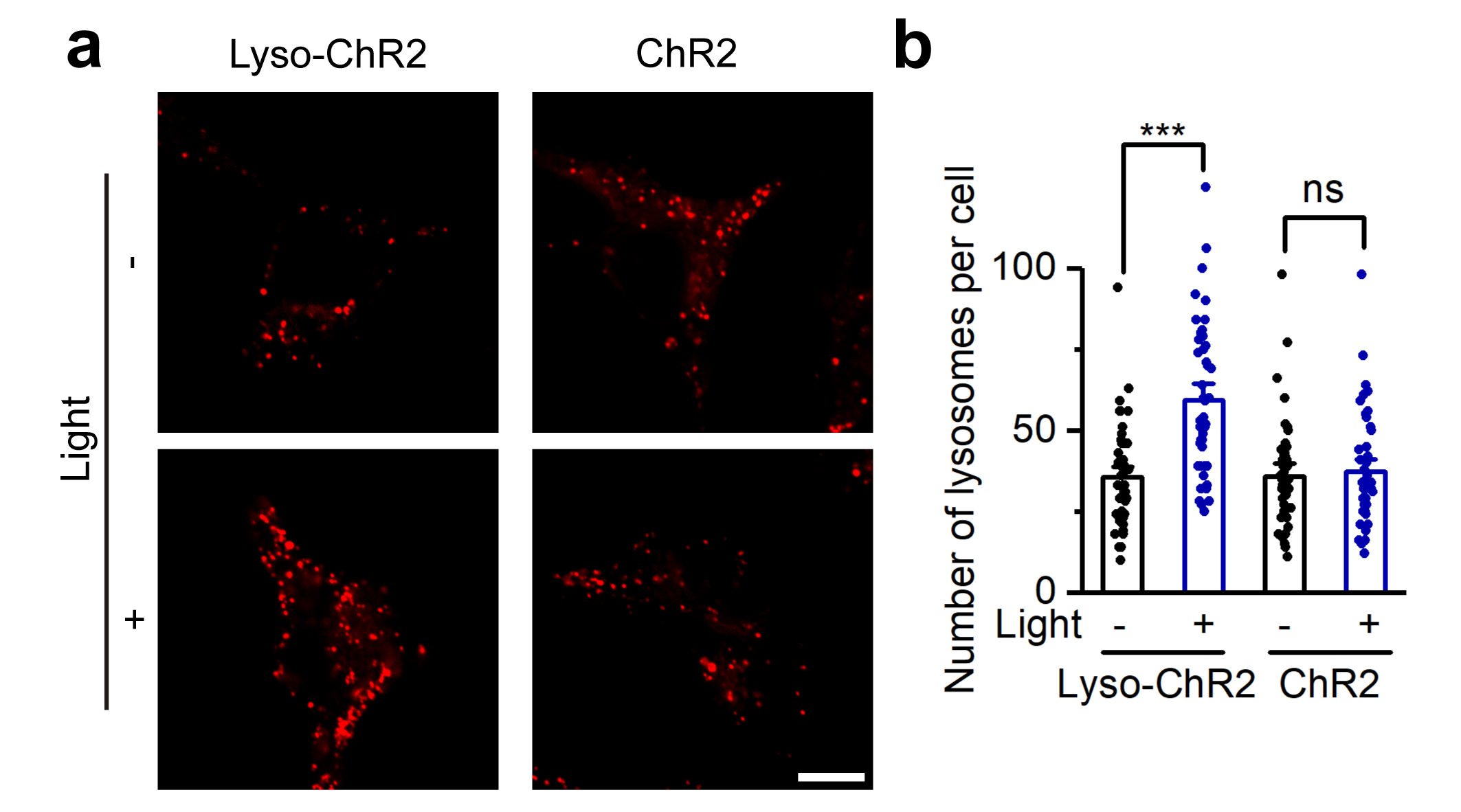

Supplement: S6 Fig — (a) Representative images of HEK293T cells stably expressing lyso-ChR2 or PM-targeted ChR2 stained with LysoTracker Red, with or without blue light. Scale bar, 10 μm. (b) Statistics of the number of lysosomes per cell in (a). n = 51, 46, 43, and 46 for bars from left to right. Data are shown as mean ± SEM. *** p < 0.001; ns, not significant. The data underlying this figure can be found in S1 Data. (TIF) [file pbio.3002591.s006.tif]

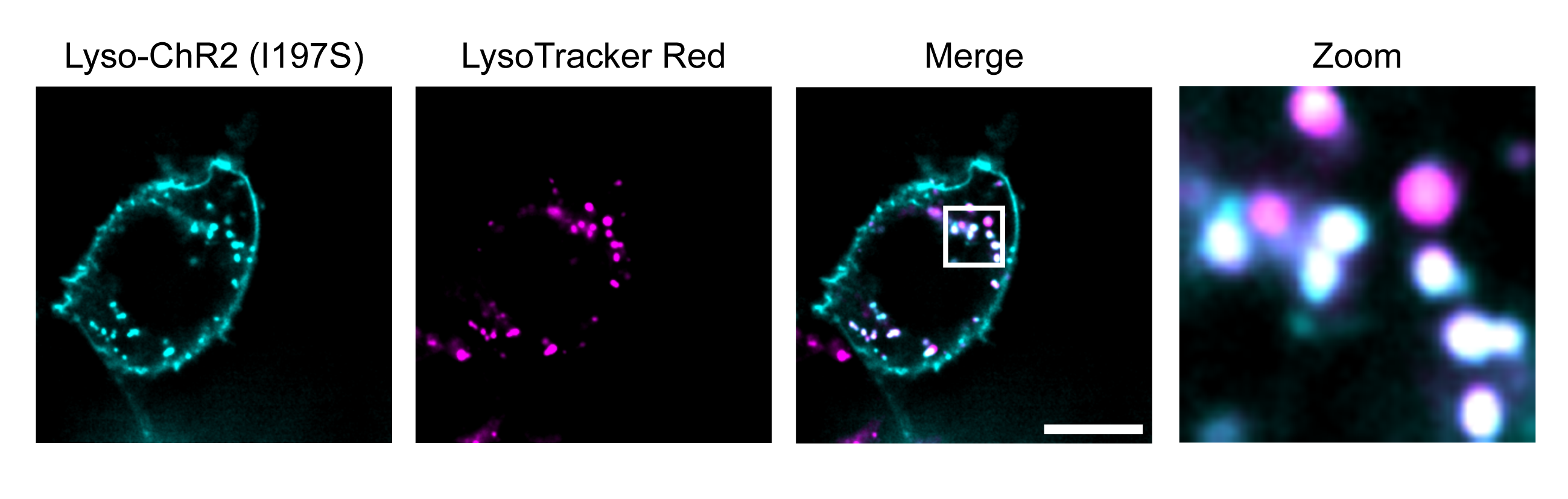

Supplement: S7 Fig — Scale bar, 10 μm. (TIF) [file pbio.3002591.s007.tif]

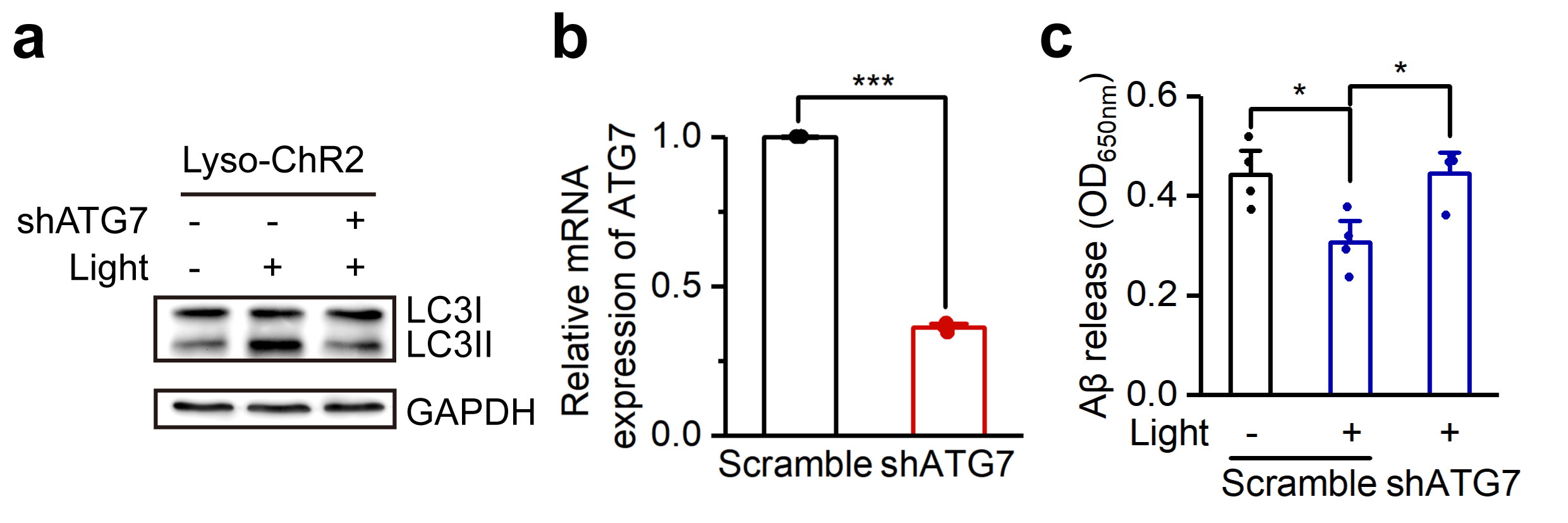

Supplement: S8 Fig — (a) The protein levels of LC3-I and LC3-II in HEK293T cells stably expressing lyso-ChR2 in response to ATG7 knockdown, with or without 6 h blue light stimulation. (b) Relative ATG7 mRNA level in lyso-ChR2-expressing HEK293T cells infected with shRNA against ATG7 (shATG7) or a scramble control. n = 3. (c) ELISA analysis of extracellular Aβ concentration in lyso-ChR2-expressing HEK293T cells in response to ATG7 knockdown, with or without 6 h blue light stimulation. n = 4. Data are shown as mean ± SEM. * P < 0.05, *** p < 0.001. The data underlying this figure can be found in S1 Data. (TIF) [file pbio.3002591.s008.tif]

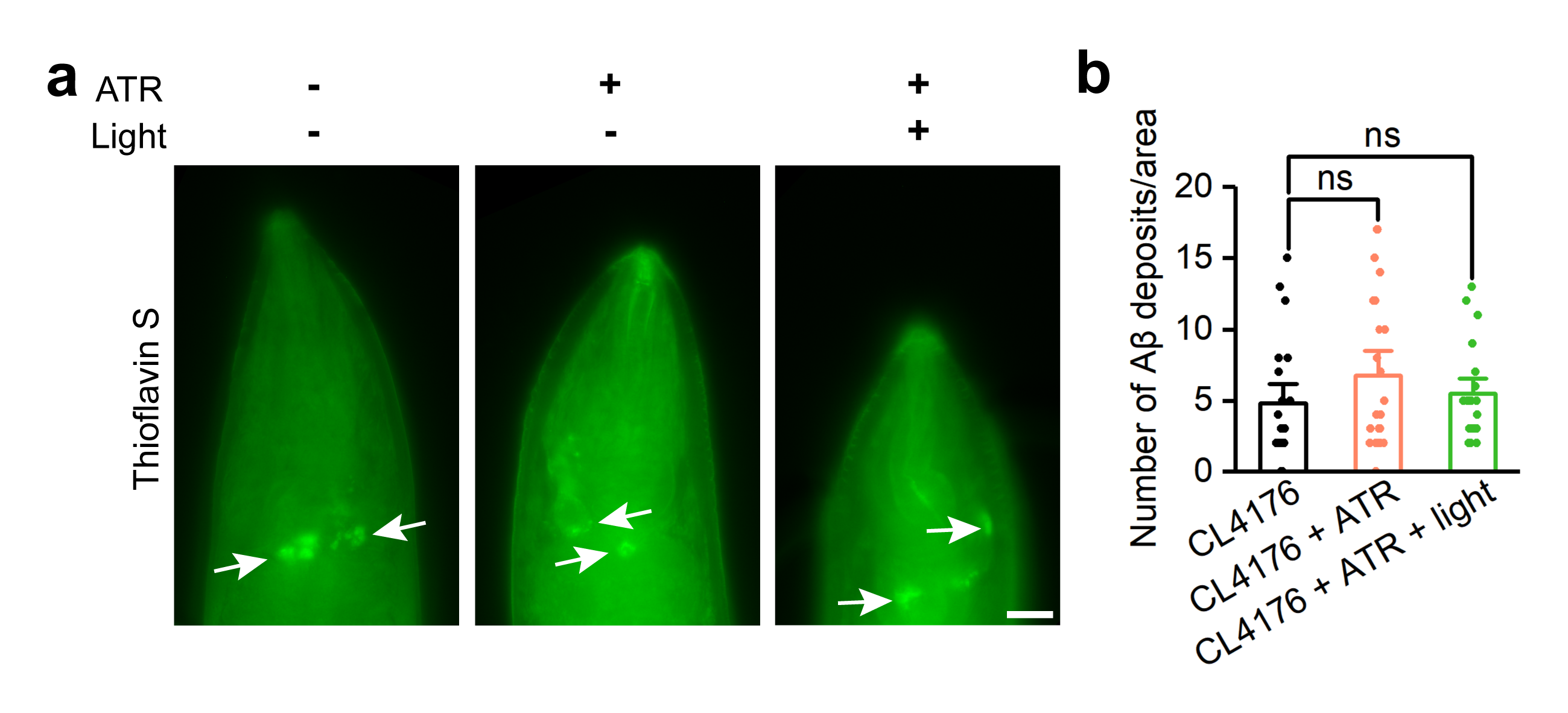

Supplement: S9 Fig — (a) Representative images of CL4176 strains stained with thioflavin S, in the presence of ATR alone or ATR with blue light exposure. Arrows indicate Aβ deposits. Scale bar, 10 μm. (b) The number of Aβ deposits in the head region/anterior area of the worms in d. n = 21, 23, and 21 worms for bars from left to right. Data are shown as mean ± SEM. ns, not significant. The data underlying this figure can be found in S1 Data. (TIF) [file pbio.3002591.s009.tif]

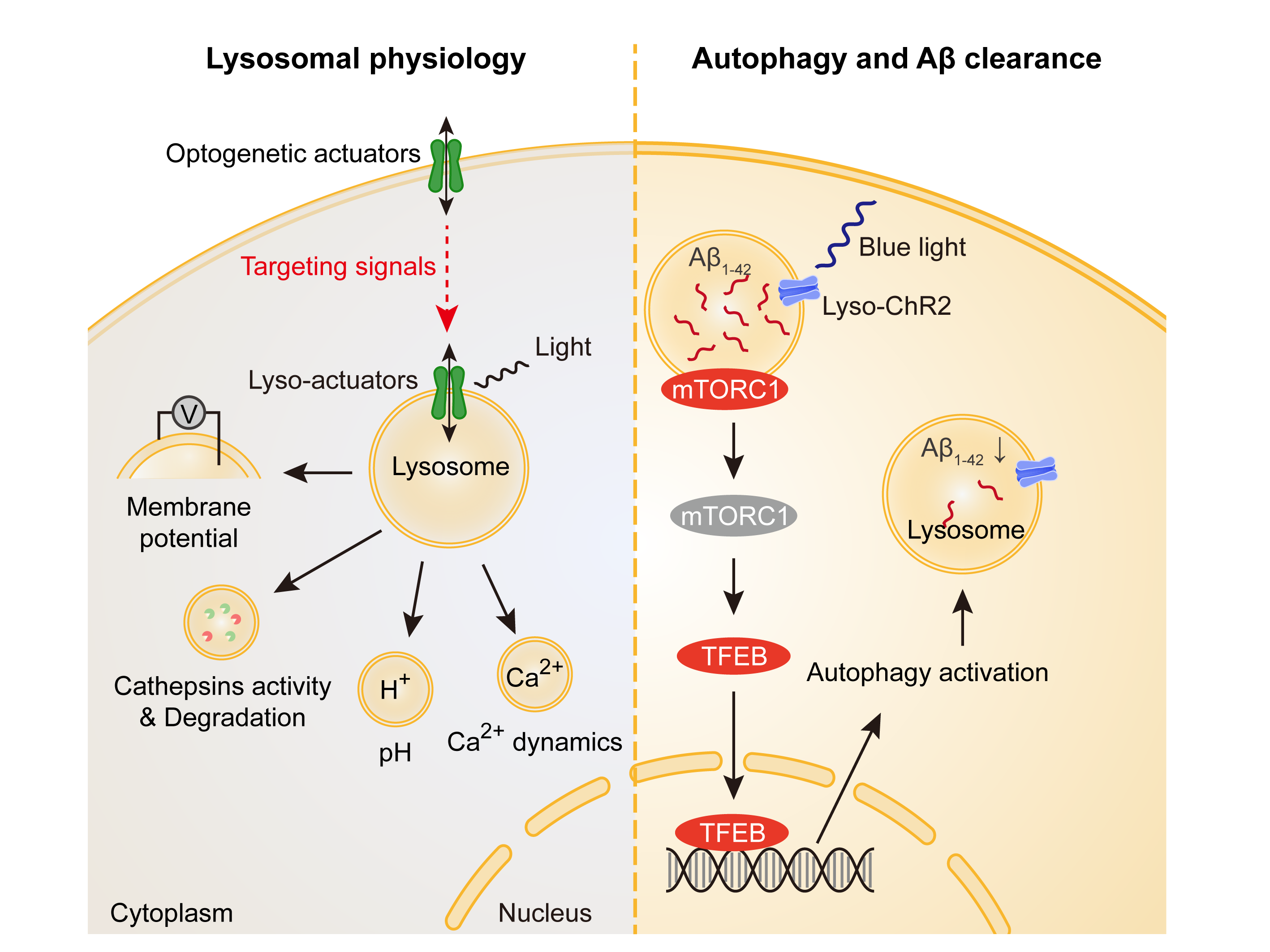

Supplement: S10 Fig — The optogenetic actuators are modified with lysosomal targeting signals to target the lysosomal membrane. Photoactivation of lysosome-targeted optogenetic actuators regulates lysosomal membrane potential, pH, Ca2+ dynamics, cathepsins activity, and degradation (left). In addition, activation of lysosome-targeted ChR2 (lyso-ChR2) induces autophagy through the mTOR pathway and enhance Aβ clearance through the autophagic pathway (right). (TIF) [file pbio.3002591.s010.tif]
