# Supplementary material for: Optogenetic manipulation of lysosomal physiology and autophagy-dependent clearance of amyloid beta
Source: PLoS Biol. 2024 Apr 23;22(4):e3002591. doi: 10.1371/journal.pbio.3002591 (PMC11068202; doi:10.1371/journal.pbio.3002591)
Supplement: S1 Raw Images — (PDF) [file pbio.3002591.s013.pdf]

S1 Raw Images: All Western blot images were captured using the ChemiDoc-It 510 Imager, and the displayed images are complete and uncropped. The uncropped source files are shown in the upper panels, the brightfield images are shown in the lower panels.

Fig 5a

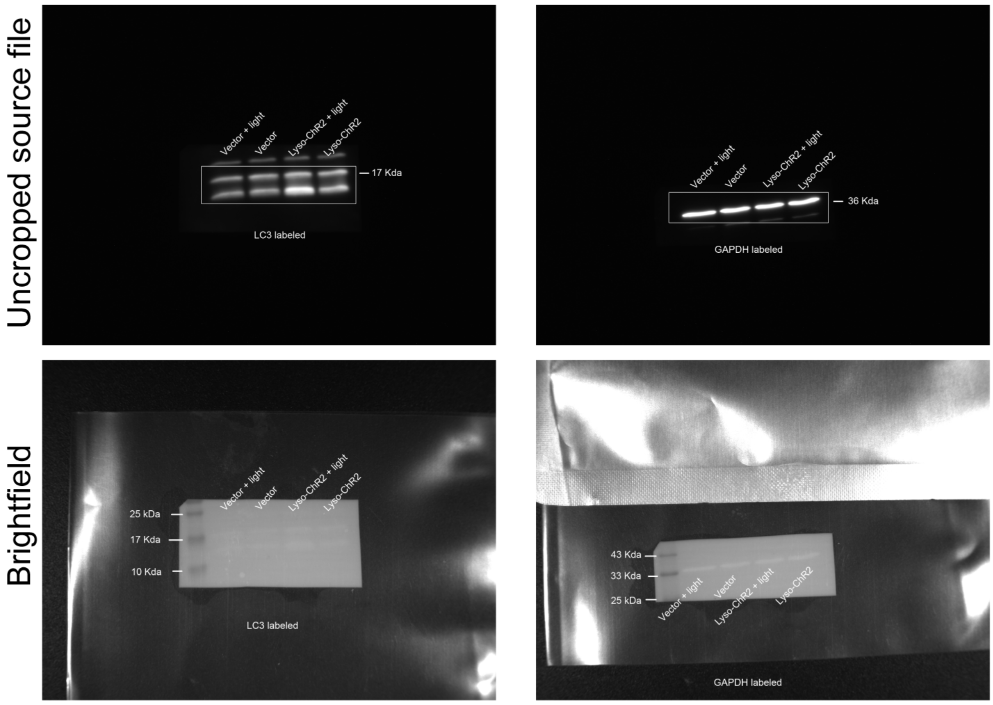

Fig 5h

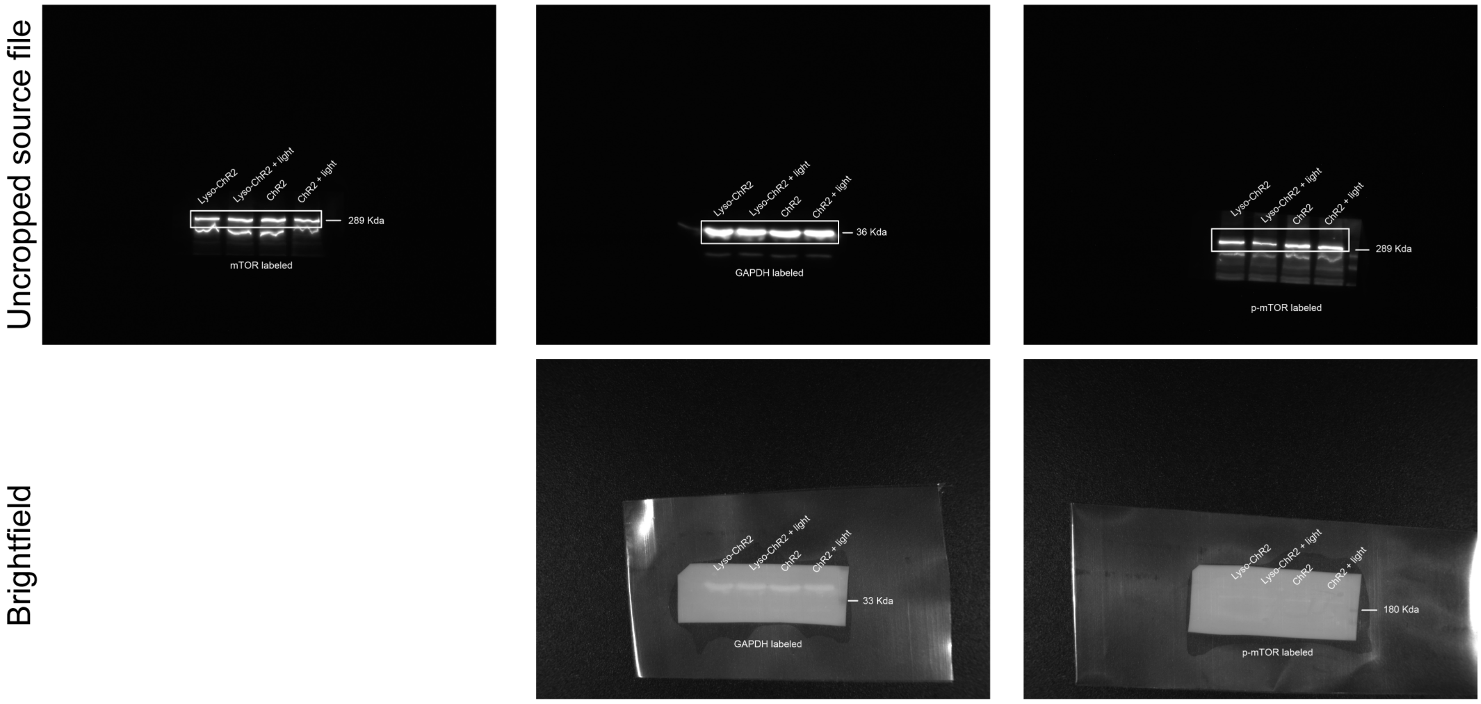

Fig 5n

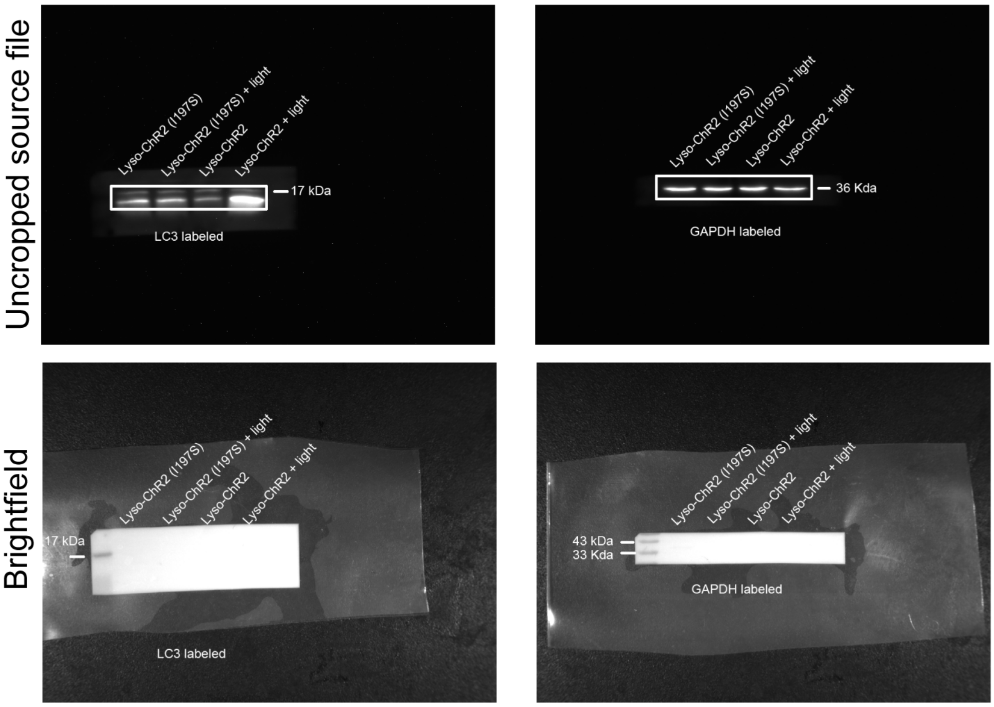
Uncropped source file
Brightfield
Lyso-ChR2 (I197S)
Lyso-ChR2 (I197S) + light
Lyso-ChR2
Lyso-ChR2 + light
17 kDa
LC3 labeled
36 Kda
GAPDH labeled
43 kDa
33 Kda

Fig 6a

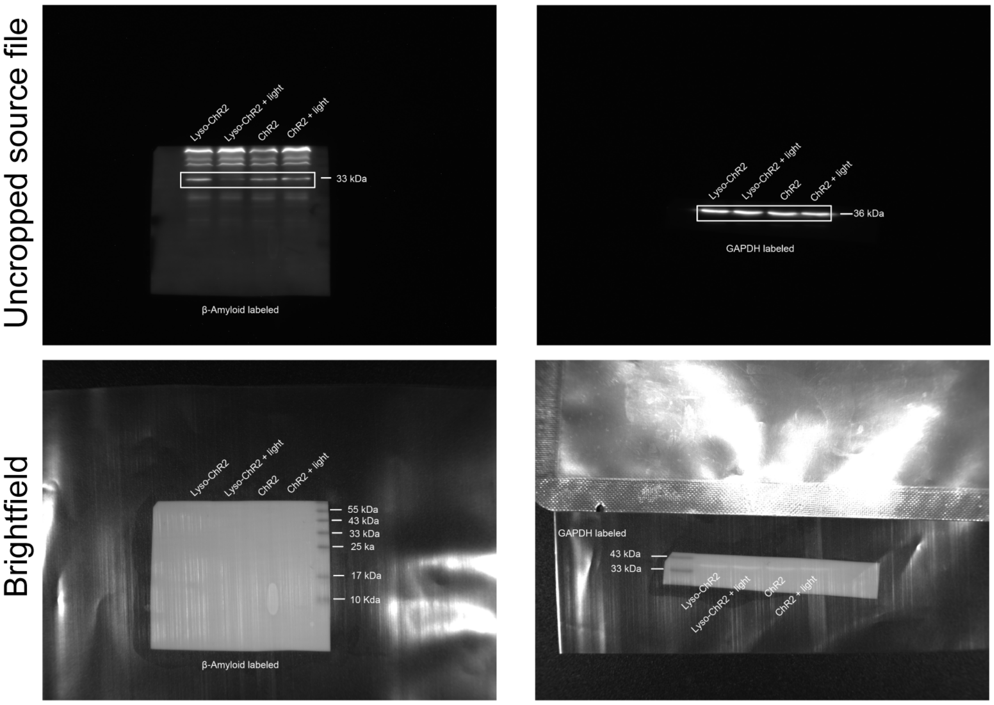
Uncropped source file
Brightfield
Lyso-ChR2
Lyso-ChR2 + light
ChR2
ChR2 + light
33 kDa
β-Amyloid labeled
36 kDa
GAPDH labeled
55 kDa
43 kDa
33 kDa
25 ka
17 kDa
10 Kda
43 kDa
33 kDa

Fig 6b

Uncropped source file

Brightfield

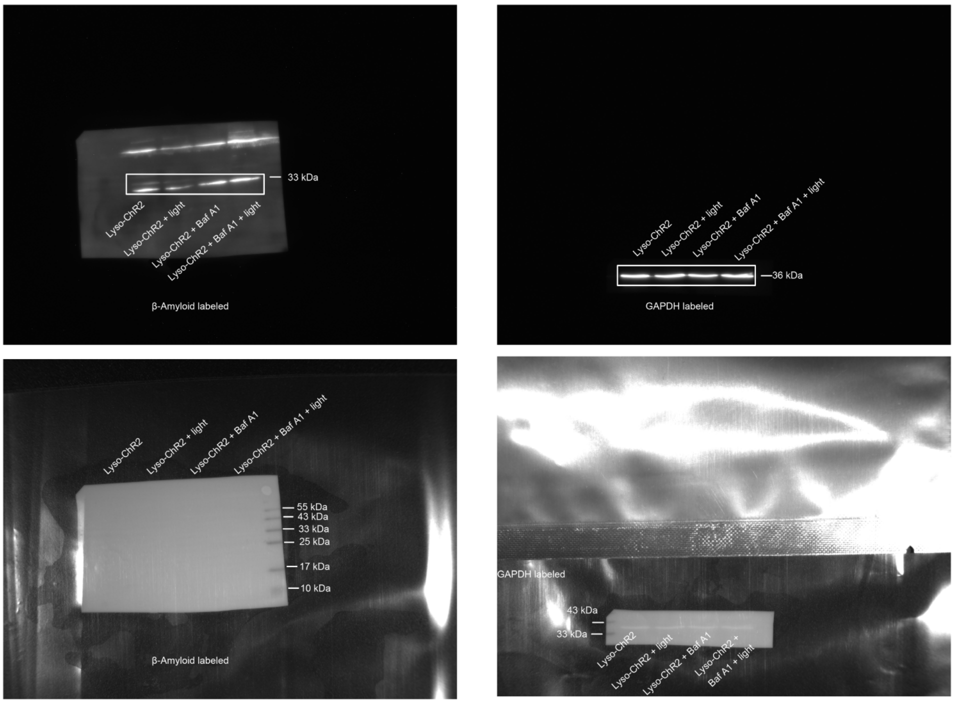

Fig 6c

Uncropped source file

Brightfield

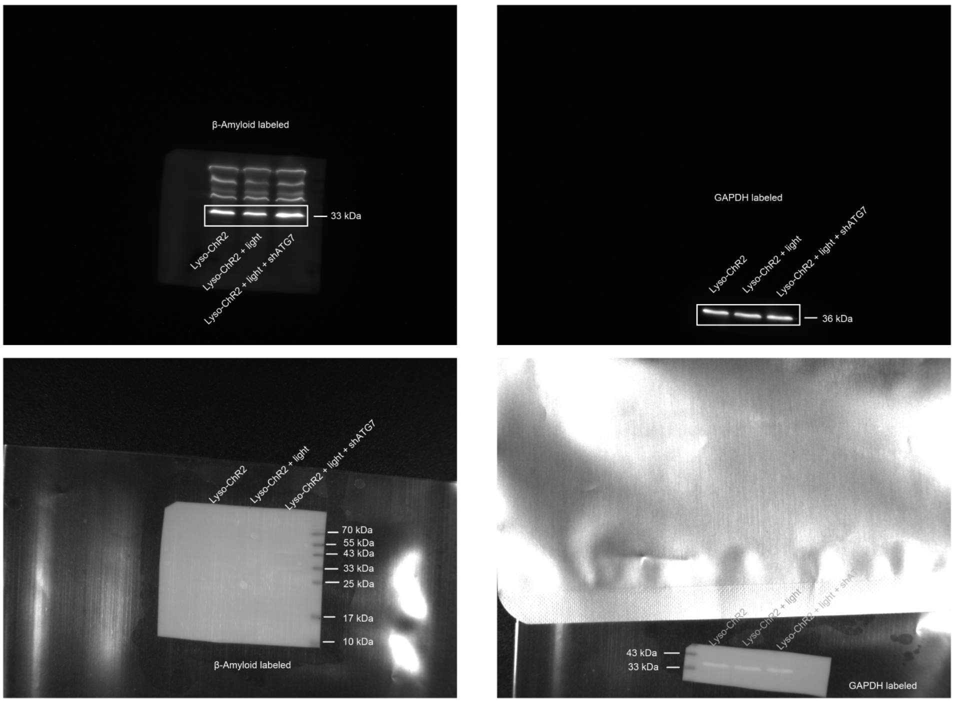

Fig S5a

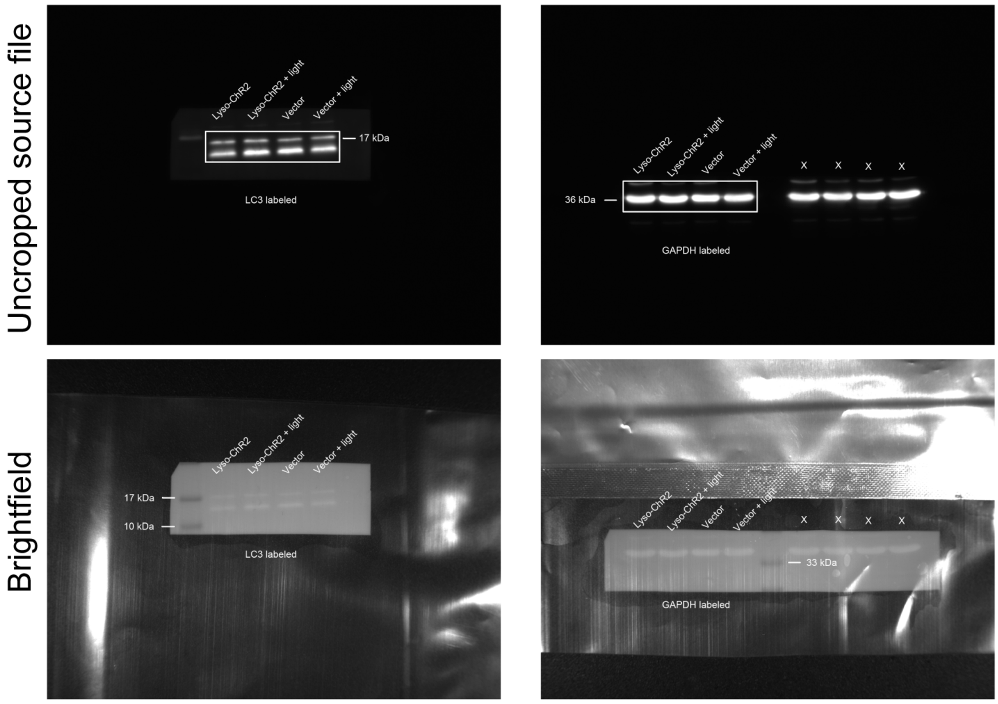

Fig S5b

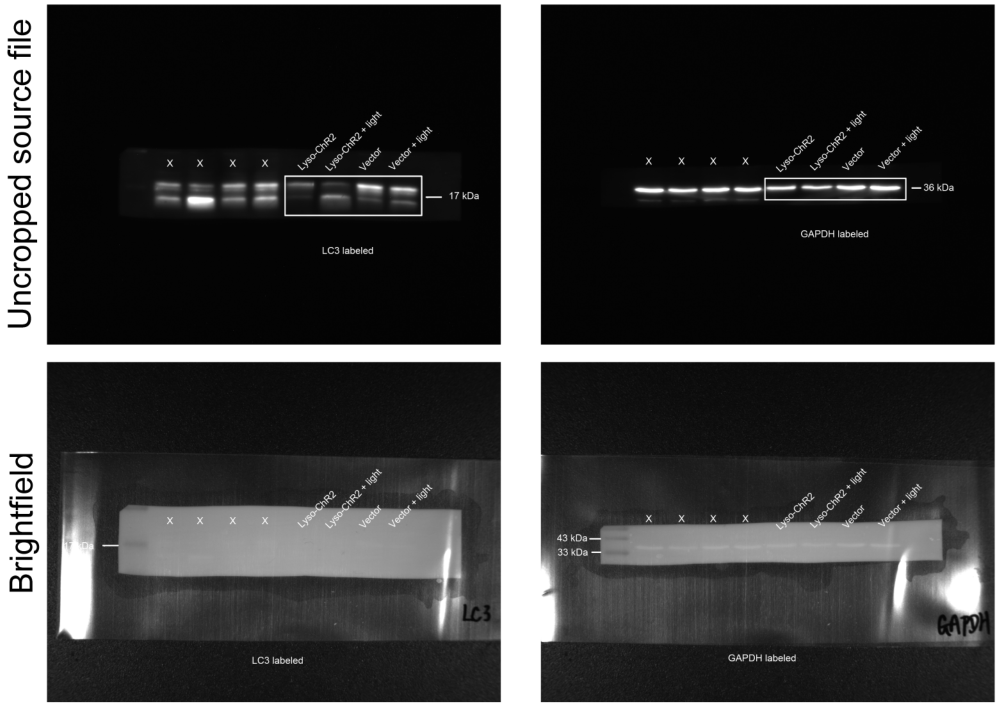

Fig S5c

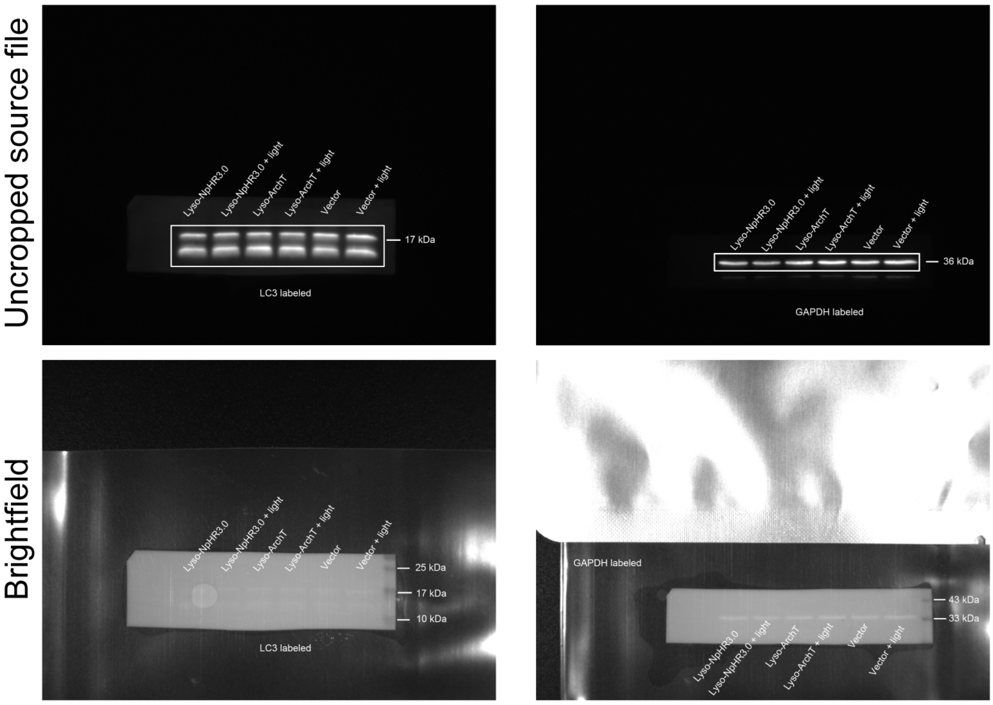

Fig S5e

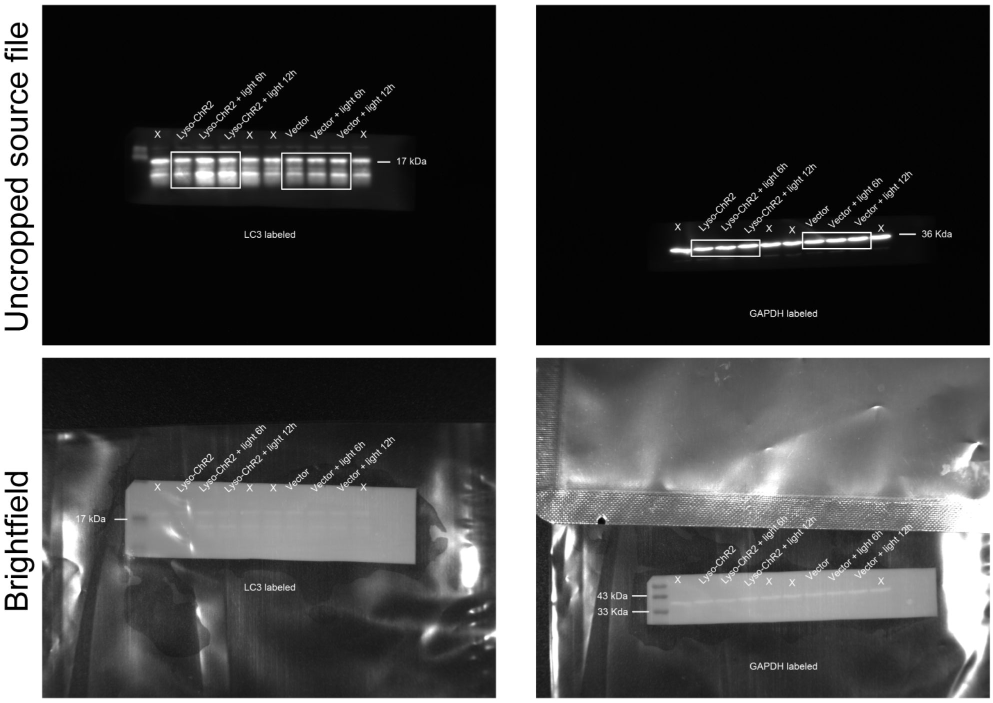

Fig S5f

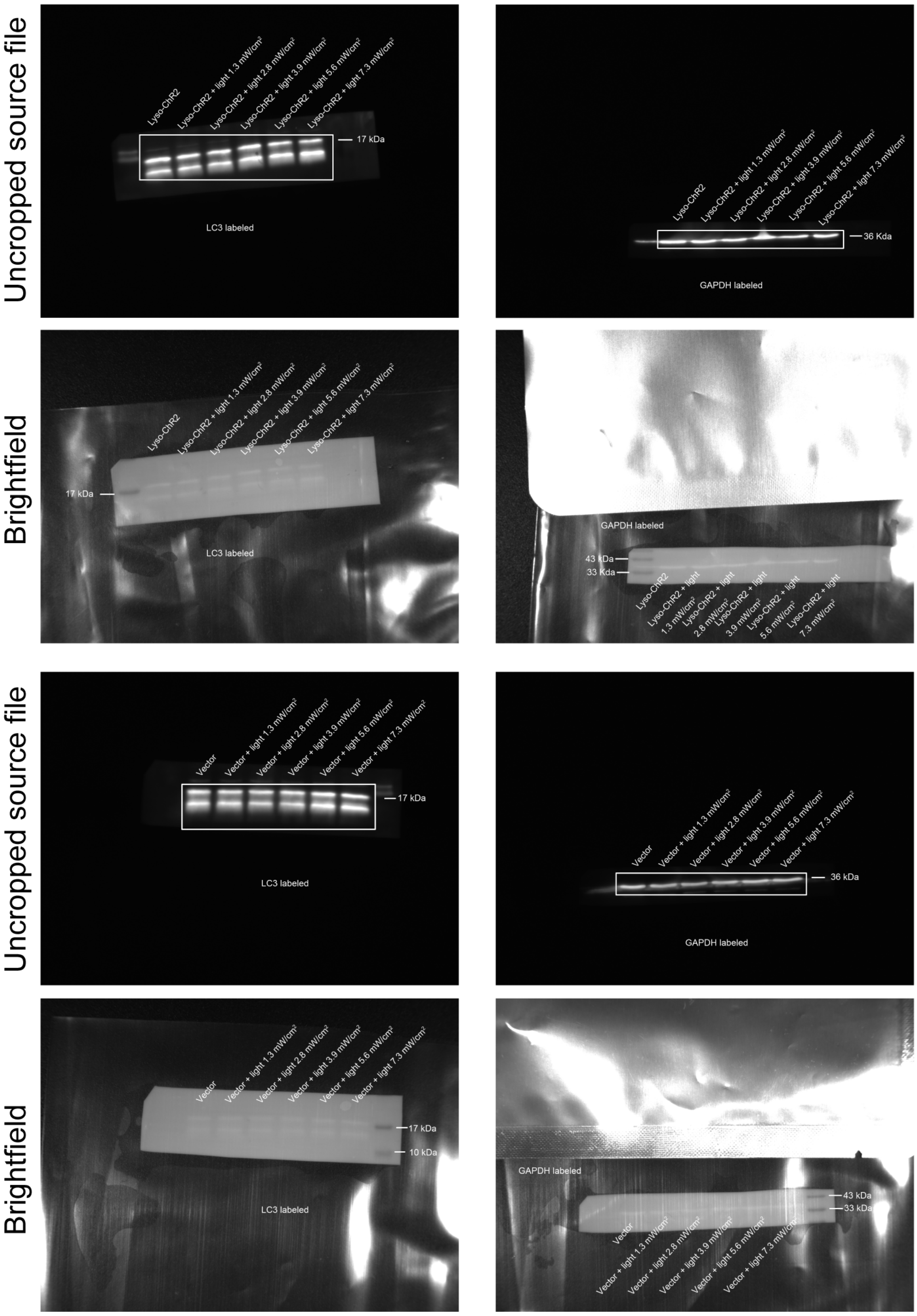
Uncropped source file
Brightfield
Uncropped source file
Brightfield
Lyso-ChR2
Lyso-ChR2 + light 1.3 mW/cm²
Lyso-ChR2 + light 2.8 mW/cm²
Lyso-ChR2 + light 3.9 mW/cm²
Lyso-ChR2 + light 5.6 mW/cm²
Lyso-ChR2 + light 7.3 mW/cm²
17 kDa
LC3 labeled
36 Kda
GAPDH labeled
43 kDa
33 Kda
Vector
Vector + light 1.3 mW/cm²
Vector + light 2.8 mW/cm²
Vector + light 3.9 mW/cm²
Vector + light 5.6 mW/cm²
Vector + light 7.3 mW/cm²
36 kDa
10 kDa
33 kDa

Fig S5g

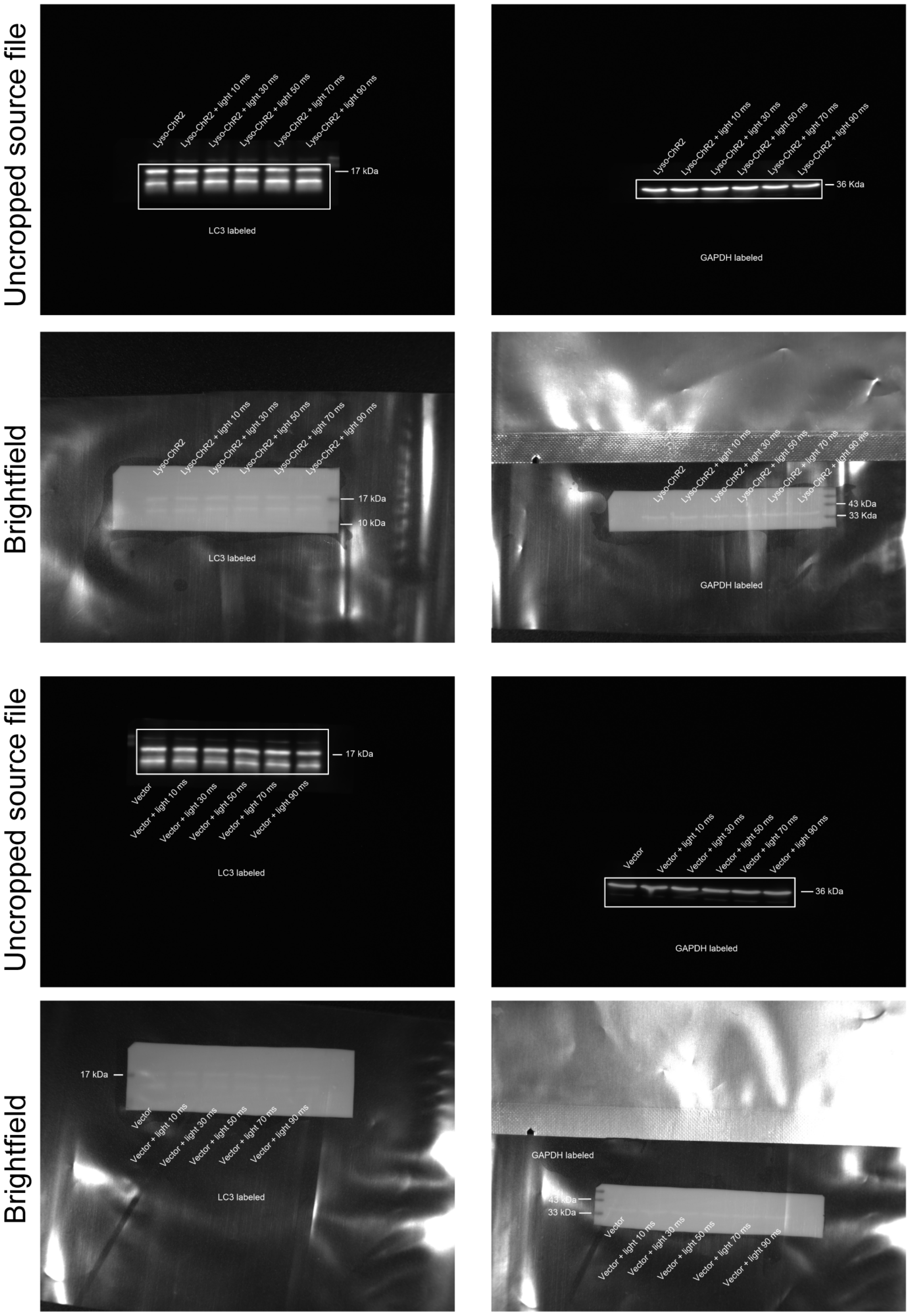
Uncropped source file
Lyso-ChR2
Lyso-ChR2 + light 10 ms
Lyso-ChR2 + light 30 ms
Lyso-ChR2 + light 50 ms
Lyso-ChR2 + light 70 ms
Lyso-ChR2 + light 90 ms
17 kDa
LC3 labeled
36 Kda
GAPDH labeled
Brightfield
17 kDa
10 kDa
LC3 labeled
43 kDa
33 Kda
GAPDH labeled
Uncropped source file
Vector
Vector + light 10 ms
Vector + light 30 ms
Vector + light 50 ms
Vector + light 70 ms
Vector + light 90 ms
17 kDa
LC3 labeled
36 kDa
GAPDH labeled
Brightfield
17 kDa
LC3 labeled
GAPDH labeled
43 kDa
33 kDa

Fig S8a

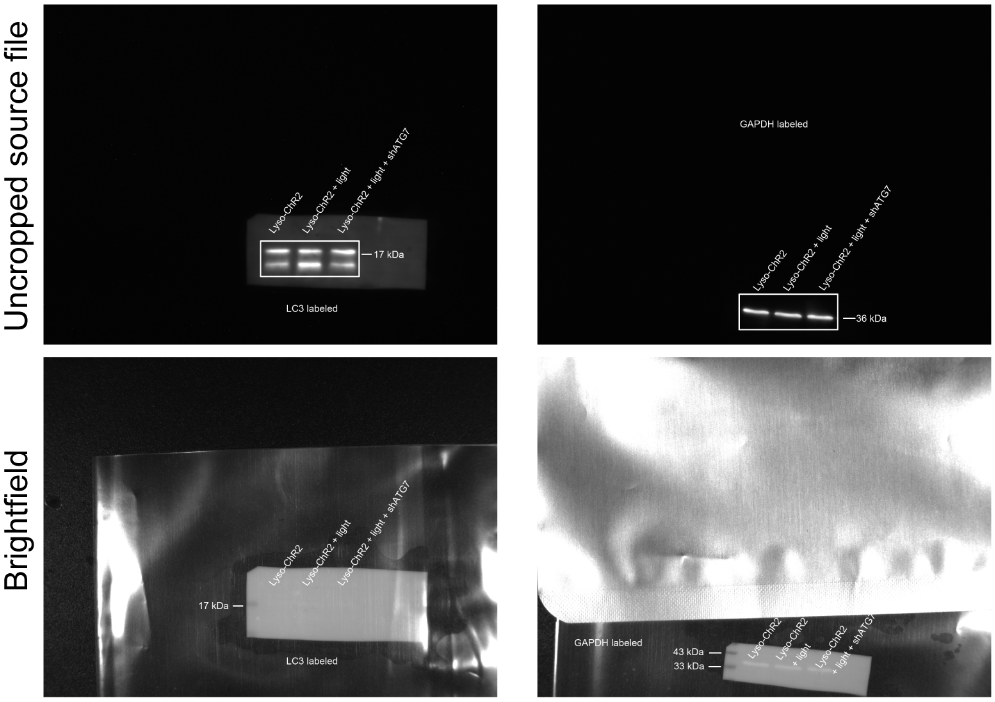

Uncropped source file
Brightfield
Lyso-ChR2
Lyso-ChR2 + light
Lyso-ChR2 + light + shATG7
17 kDa
LC3 labeled
GAPDH labeled
Lyso-ChR2
Lyso-ChR2 + light
Lyso-ChR2 + light + shATG7
36 kDa
Lyso-ChR2
Lyso-ChR2 + light
Lyso-ChR2 + light + shATG7
17 kDa
LC3 labeled
GAPDH labeled
43 kDa
33 kDa
Lyso-ChR2
Lyso-ChR2 + light
Lyso-ChR2 + light + shATG7
